# ﻿*Capparis* (Capparaceae) in Peninsular Malaysia, including a new species and two new varieties

**DOI:** 10.3897/phytokeys.189.49367

**Published:** 2022-02-04

**Authors:** Avelinah Julius

**Affiliations:** 1 Forest Research Institute Malaysia, Kepong, Selangor, 52109, Malaysia Forest Research Institute Malaysia Kepong Malaysia

**Keywords:** Brassicales, conservation, endemics, key, Peninsular Malaysia, sect. *Monostichocalyx*, taxonomy

## Abstract

As part of the Flora of Peninsular Malaysia Project, a species checklist of the genus *Capparis* in Peninsular Malaysia is presented here with a total of 11 species, two subspecies and four varieties. A new species and two varieties, endemic to Peninsular Malaysia, are described and illustrated: *Cappariskenaboiensis*, C.scortechiniivar.ruthiae and C.trinerviavar.chungiana. *Cappariskenaboiensis* is known from a single site in Negeri Sembilan and is assessed as Vulnerable (VU); C.scortechiniivar.ruthiae from Pahang is Vulnerable (VU); and Capparistrinerviavar.chungiana is known from Negeri Sembilan, Pahang and Selangor and its conservation status is assessed as Near Threatened (NT). A checklist and updated key to the genus in Peninsular Malaysia are provided.

## ﻿Introduction

*Capparis* L., comprising from 130 to 141 species, is the largest genus in Capparaceae (POWO 2019; Souvannakhoummane et al. 2020) and distributed in the tropical and subtropical regions of the Old World. The genus occupies a wide range of habitats, such as forest margins, coastal vegetation, rocky vegetation, savannahs and thickets. Four sections are usually recognised, viz. Capparissect.Capparis, sect. Busbeckea (Endl.) Benth. & Hook.f. (Bentham and Hooker 1862:109), sect. Monostichocalyx Radlk. (Radlkofer 1839: 101) and sect. Sodada (Forsk.) Endl. (Endlicher 1839: 893). Of these four sections, sect. Monostichocalyx is further subdivided into seven groups based on inflorescence characters: Brevispina-, Cataphyllosa-, Grandis-, Moonii-, Seriales-, Subumbellates- and Trinervia-Group (Jacobs 1965).

Generic delimitation and relationships amongst *Capparis* species using morphological and molecular approaches have shown the New World and Old World species of *Capparis* do not form a monophyletic group (Hall 2008; Cardinal-McTeague et al. 2016; Tamboli et al. 2018). The New World taxa, traditionally identified as *Capparis* s.l., differ from Old World *Capparis* by the following traits: absence of prickles, symmetry of flowers, calyx types, calyx and corolla aestivation, different floral nectary structures and different fruit and embryo types (Cornejo and Iltis 2009a). Thus, all species of *Capparis* in the Neotropics belong to other genera, with the species now transferred into several genera (e.g. Cornejo and Iltis 2008, 2009a, 2009b, 2013; Cornejo 2017).

In Peninsular Malaysia, at least ten species and two subspecies were known (Jacobs 1960, 1965; Turner 1995; Kiew et al. 2009) - all members of sect. Monostichocalyx, characterised by the well-developed persistent leaves, sepals free in bud and dimorphic in which the outer sepals are larger in size, enclosing the narrower inner pair of sepals (Jacobs 1960: 410). These members were placed into four groups within this section: *Capparisscortechinii* King and *C.trinervia* Hook.f. & Thoms. in the Trinervia-Group; *C.diffusa* Ridl., *C.erycibe* Hallier f., *C.sepiaria* L. and *C.versicolor* Griff. in the Subumbellates-Group; *C.pubiflora* DC. in Cataphyllosa-Group; and *C.acutifolia* Sweet, *C.cucurbitina* King and *C.micracantha* DC. in the Seriales-Group. Of these ten species, *C.cucurbitina* was the only endemic to Peninsular Malaysia, being known from Gunung Bubu, Perak.

During a study of *Capparis* specimens for revisionary work on the genus for the Flora of Peninsular Malaysia account, several specimens which were determined as previously-described species (following Jacobs 1965) were critically re-evaluated as two undescribed taxa. These two are clearly distinct in several of their vegetative and reproductive characters from any known species. Another taxon discovered during the 2014 expedition to Kenaboi Forest Reserve (FR), Negeri Sembilan, also represents an undescribed taxon. After morphological comparison with closely related species (see below) and consulting the relevant literature (Jacobs 1960, 1965; Chayamarit 1991; Srisanga and Chayamarit 2004; Zhang and Tucker 2008; Fici 2012, 2016, 2021; Thuong et al. 2013, 2015, 2016, 2017; Fici et al. 2018; Souvannakhoummane et al. 2018; Fici et al. 2020; Fici and Souvannakhoummane 2020; Souvannakhoummane et al. 2020), these taxa are here recognised as three new taxa. Subsequently, in the checklist presented here, there are 11 species of *Capparis* in Peninsular Malaysia, with two subspecies and four varieties; all taxa are members of sect. Monostichocalyx. In addition to these three new taxa, Peninsular Malaysia now has four endemic taxa of *Capparis*. A key is provided to facilitate identification of the species.

## ﻿Materials and methods

Morphological observations were made from herbarium material held at BKF, K, KEP, KLU, L, SAN and SING. Specimens deposited in CAL were studied, based on scanned images. Additionally, specimen images from Global Plants JSTOR (http://plants.jstor.org/), Conservatoire & Jardin botaniques de la Ville de Genève. (http://www.ville-ge.ch/musinfo/bd/cjb/chg) and the BioPortal of Naturalis Biodiversity Center (http://bioportal.naturalis.nl/) were consulted. Floral measurements were made from either rehydrated or fresh material; details are given, based on specimen labels. Flowering and fruiting materials are indicated by ‘fl.’ and ‘fr.’, respectively, under the specimens examined section for the new taxa. Specimens were mapped and a conservation assessment of the species undertaken using the IUCN Red List Categories and Criteria (IUCN 2001, 2012) following the guidelines and procedures developed at FRIM for the Malaysia Plant Red List (Chua and Saw 2006).

## ﻿Taxonomic treatment

### ﻿*Capparis* L.

Shrubs, often sprawling or climbing, rarely small trees. ***Twigs*** flexuous or straight. ***Indumentum*** mostly simple hairs, sometimes stellate, often glabrescent. ***Stipular thorns and/or prickles*** straight or retrorse, divaricate, often well-developed and persistent on main branches, sometimes rudimentary (rarely lacking). ***Leaves*** alternate or spirally arranged; lamina simple, coriaceous, subcoriaceous or chartaceous, sometimes extra membranous, margin entire, edges flat or recurved, lateral veins ascending regularly, sometimes brochidodromous interlooping near the margin. ***Inflorescence*** axillary, lateral or terminal; bracts mostly present, but early caducous, rarely to basal bracteoles. ***Flowers*** solitary or fascicle in the axils or in a series with 2–6-flowered along the twig or arranged in corymbs, umbels or subumbels or paniculate; sepals free in bud, 4, arranged in pairs and opposing sepals equal in size, imbricate or not; petals 4, asymmetrical, the dorsal petals erect and laterally connate at base, lateral ones spreading and free, rather delicate; stamens 6 to numerous, filaments of unequal length, anthers tetrasporangiate, basifixed or dorsifixed, opening lengthwise; ovary 1-locular, on gynophores as long as the stamens or longer, not or very little stretching in fruit, stigma obscure or small, sessile (elsewhere and in Peninsular Malaysia) or in capitate or cushion-shaped (elsewhere, but not in Peninsular Malaysia). ***Fruit*** an amphisarca with a subwoody (when young) turning pulpy (when ripe) exocarp, indehiscent (elsewhere and in Peninsular Malaysia) with the exception of *Cappariscartilaginea* Decne. and *C.spinosa* L. (a capsular: ripened fruit dehiscent longitudinally), globose, ellipsoid or torulose, exocarp smooth with or without longitudinal ribs (fruit is without longitudinal ribs in Peninsular Malaysia) or tuberculate (elsewhere, but not in Peninsular Malaysia). ***Seeds*** few to many rarely 1; embryo tightly coiled with the cotyledons in the centre; cotyledons stipulate, ovate or elliptic, white.

### ﻿Groups of sect. Monostichocalyx

Capparissect.Monostichocalyx, distributed from Africa (except the northern part) to Asia, extended to Australia and the Pacific, contains the majority of species in the genus with ca. 110 taxa (Jacobs 1965; Srisanga and Chayamarit 2004; Fici 2012, 2021; Thuong et al. 2013, 2015, 2016, 2017; Fici et al. 2018; Souvannakhoummane et al. 2018). Members of this section have been divided into seven groups, but Peninsular Malaysian taxa fall into four groups as mentioned earlier in introduction.

### ﻿A synoptic key to the four groups of C.sect.Monostichocalyx in Peninsular Malaysia (adopted and modified from Jacobs 1965)

**Table:** 

1c	Flowers large (sepals 8–15 mm long, torus ca. 5 mm wide), arranged in corymbs, umbels to subumbels or racemes. Plants with brown hairs. Twigs angular when young, with mostly strong, recurved stipular thorns and/or prickles. Leaves with obscure nerves, dull greenish with brown-coloured nerves, longer than 6 cm. Stamens 30–75	**III. Trinervia-Group**
1d	Flowers more or less neatly subumbellate, the subumbels axillary and/or arranged to panicles. Flowers medium-sized to small (sepals 2–10 mm long). Stamens < 70. Ovary 1–3 mm long	**IV. Subumbellates-Group**
1f	Flowers large to small (sepals 3–14 mm long), arranged on a long or short leafless bracteates axis or subumbellate. Shoots with cataphylls at the base. Hairs 2-armed or hooked or acroscopically curved. Stipular thorns usually ascending. Leaves mostly over 8 cm long, mostly dull	**VI. Cataphyllosa-Group**
1g	Flowers mostly small to medium-sized (sepals 3–15 mm long), serial in supra-axillary rows (or sometimes small bundles of cataphylls in their place), exceptionally all solitary, but in that case, the twigs red-hairy and the leaves shorter than 3.5 cm. Indumentum, if present, often (red-)brownish	**VII. Seriales-Group**

### ﻿Key to the *Capparis* species in Peninsular Malaysia

(Based on flowering/fruiting specimens)

**Table:** 

1	Flowers arranged in a series along the twig just above the leaf axil with up to 6 flowers	**2**
–	Flowers arranged in a short fascicle, umbels to subumbels, racemes or paniculate, either terminal on leafy twigs or axillary in the leaf axil	**5**
2	Stipular thorns absent	***C.acutifolia***
–	Stipular thorns present	**3**
3	Leaf apex long acuminate, 10–13 mm long. Petals broadly elliptic. Fruit torulose	***C.cucurbitina***
–	Leaf apex mucronate or shortly acuminate, 4–6 mm long. Petals oblanceolate or obovate to elliptic. Fruit ellipsoid or oblong	**4**
4	Leaf subcoriaceous to chartaceous, base cordate, apex mucronate. Petals up to 1.6 cm long. Stamens 15–18	**C.micracanthasubsp.micracantha**
–	Leaf coriaceous, base cuneate, apex shortly acuminate. Petals up to 2.4 cm long, stamens > 18	**C.micracanthasubsp.korthalsiana**
5	Inflorescences in axillary fascicles	***C.pubiflora***
–	Inflorescences in terminal and/or axillary racemes, paniculate, umbels or subumbels	**6**
6	Inflorescences paniculate	***C.erycibe***
–	Inflorescences racemose, umbels or subumbels	**7**
7	Inflorescences strictly racemose, flowers densely or loosely arranged in raceme	**8**
–	Inflorescences umbellate, subumbellate and/or flowers arranged racemosely and becoming crowded at the distal part of the inflorescence	**10**
8	Flowers loosely arranged in racemes with early caducous leaf-like bracts. Lamina surface bullate	***C.kenaboiensis* sp. nov.**
–	Flowers densely arranged in racemes and subtended by persistent and conspicuous leaf-like bracts. Lamina surface smooth	**9**
9	Racemes axillary and/or terminal. Leaf-like bracts thick, densely hairy with velvety, shiny and rusty hairs abaxially, glabrous adaxially, elliptic, 25–30 × 10–13 mm. The gynophore densely hairy at base	**C.scortechiniivar.scortechinii**
–	Racemes strictly terminal. Leaf-like bracts thin, densely hairy with tomentose, straw hairs on both surfaces, soon glabrescent abaxially with age, narrowly elliptic, (10–)13–25 × (1–)2–8 mm long. The gynophore glabrous throughout	**C.scortechiniivar.ruthiae var. nov.**
10	Inflorescences in racemes and/or subumbels, terminal sometimes axillary, sepals hairy	**11**
–	Inflorescences in umbels, lateral or axillary sometimes terminal, sepals glabrous	**12**
11	Lamina subcoriaceous to coriaceous, broadly ovate sometimes ovate-elliptic, 13–16 × 5.5–8.5 cm, drying leaves reddish brown rarely pale green with pale yellow rarely dark red venation on both surfaces, intercostal veins obscure. Inflorescence terminal with flowers arranged racemosely and becoming crowded at the distal part of the inflorescence, stamens 30–40	**C.trinerviavar.chungiana var. nov.**
–	Lamina chartaceous, oblong-elliptic or broadly lanceolate, (5–)10–14(–19) × (2–)3.5–8.5 cm, drying leaves dull green with brownish main nerves on both surfaces, intercostal veins irregular reticulate and distinct. Inflorescence terminal with flowers arranged racemosely and becoming crowded at the distal part of the inflorescence, sometimes subumbellate on 3–4 cm long peduncles in the axils of the uppermost leaves, stamens (30–)60–70	**C.trinerviavar.trinervia**
12	Twigs obviously flexuous	***C.sepiaria***
–	Twigs ± straight	**13**
13	Leaf < 5 cm long, margin revolute, lamina coriaceous to subcoriaceous, apex obtuse or retuse. Umbels pedunculate, 1–4-flowered, axillary and/or terminal	***C.versicolor***
–	Leaf > 5 cm long, margin flat at the edge and not revolute, lamina chartaceous, apex usually obtuse sometimes acute, with acumen 5–10 mm long. Umbels sessile, 3–5-flowered with 1–2 small leaves, sometimes a few umbels united to a small panicle, terminal or lateral on small twigs	***C.diffusa***

### ﻿New taxa

#### ﻿ Capparis kenaboiensis

Taxon classificationPlantaeBrassicalesCapparaceae

Julius sp. nov.

BD97B648-DB8E-5EE0-92CD-99438639F6EE

urn:lsid:ipni.org:names:77254614-1

Figures 1 , 2 , 3

##### Diagnosis.

Vegetatively, this new species resembles *Capparisbuwaldae* Jacobs in having bullate leaves with a long acuminate-caudate apex and distinct reticulation, but *C.kenaboiensis* differs from *C.buwaldae* in its terminal (vs. supra-axillary) inflorescences, the absent (vs. present) stem stomata and the smooth (vs. tuberculate) fruit pericarp. The flowers subtended by leaf-like bracts resemble *Capparisscortechinii*, but are early caducous in the new species.

##### Type.

Peninsular Malaysia. Negeri Sembilan: Jelebu, Kenaboi FR, Gunung Besar Hantu, road sides towards Lata Kijang, 3°11.42'N, 100°59.21'E, 459 m alt., 4 Mar 2014 (fl., fr.), *Julius et al. FRI57784* (holotype KEP!; isotypes K!, SAN!, SING!, L!, A!).

##### Description.

Climber 2–8(–12) m long hanging high up on tree. ***Twigs*** straight, pubescent when young and soon glabrous. ***Stipular thorns*** retrorse, tips 1–2 mm long. ***Leaves*** spirally arranged; petioles 8–12 × ca. 1 mm, grooved, slender, not thickened, covered with dense, short, silky white hairs; laminas narrowly elliptic, (5.5–)10–11.5 × (1.5–)3.5–4 cm, chartaceous, bullate, fresh dark green and glossy above, pale green beneath, brownish-green when dry, base acute, margin revolute and entire, apex long acuminate to caudate, with acumen 1–1.5 cm long, glabrous above, hairy beneath, denser on mid-rib and venation; mid-rib sunken above, raised beneath; lateral veins 5–7 pairs, arcuate towards the margin; intercostal veins reticulate, distinct above, prominent beneath. ***Inflorescences*** axillary or terminal, racemose, rachis slender and flexuous, conferted towards the top as the buds fall off at bottom part and leave scars, velvety, white hairs throughout; bracts leaf-like, elliptic, 10–25 × 2–5 mm, velvety white hairs on both surfaces, early caducous. ***Flowers*** loosely arranged, 10–14, buds globose, 4–5 × 3–5 mm; pedicels 2–2.5 cm long, whitish; sepals 4, thin, cucullate, whitish-green, except reddish at base inside, keeled, 2 pairs, lanceolate, 6–8 × ca. 5 mm, outer pair larger than inner pair, the outer sepals imbricate, covered with dense, white hairs outside, glabrous inside, the inner pair hairy on keel outside, glabrous inside; petals 4, upper pair pinkish and white along margin and laterally connate at base, lower pair whitish-green and free, elliptic ca. 10 × 3 mm, inside densely, silky tomentose hairy, outside glabrous, except densely hairy at base, ciliate along the margin; stamens 18–31, unequal in length, filament whitish, 7–20 mm long, glabrous, except hairy at base, anthers dorsifixed, ca. 1.5 mm long, greenish, apex recurved; ovary ellipsoid, ca. 1.5 mm long, greenish, on gynophore 2–2.5 cm long, stigma obscure. ***Fruits*** young green turning brownish to dark purple when ripe, subglobose to pyriform on slender gynophore, 4.5–5 × 5.5–6 cm, pulp pinkish or purplish-red. ***Seeds*** 1–4, sarcotesta fleshy, yellowish, testa thin and black.

##### Distribution.

**Figure 1.**
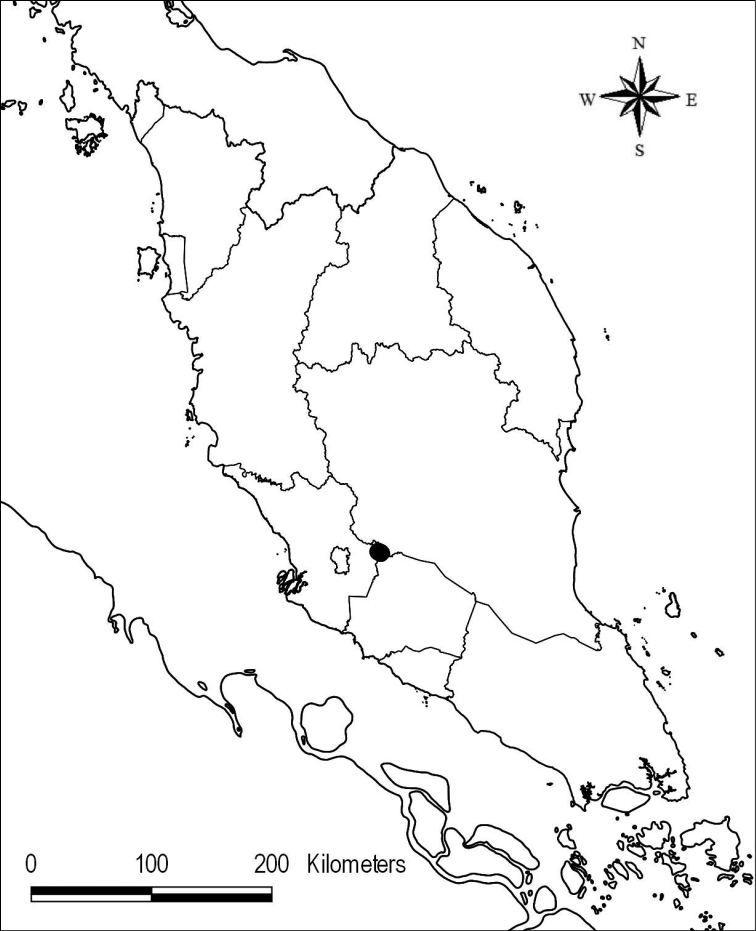
Distribution of *Cappariskenaboiensis*.

Endemic in Peninsular Malaysia and known only from the type locality (Fig. 1).

##### Ecology.

Secondary forest margin, in light shade.

##### Conservation status.

*Cappariskenaboiensis* is known from a single locality and is very rare with less than ten individuals found in two subpopulations. The species inhabits a secondary forest margin and by a pathway, which are vulnerable to forest clearing pathways as was observed in 2014 during the botanical survey. Moreover, only two sub-populations were observed during a recent visit in 2019. As the area of occupancy is less than 500 km^2^ and the declining of habitat, this species is assessed as Vulnerable B2ab(iii), following the IUCN Red List Categories and Criteria (IUCN 2001, 2012).

##### Additional specimens examined.

**Peninsular Malaysia. Negeri Sembilan**: Jelebu, Kenaboi FR, G. Besar Hantu, from Taman Alam Liar towards Trail 1, near pathways, 3°12'N, 100°58'E, 530 m alt., 4 Mar 2014 (fl., fr.), *Julius et al. FRI57797* (KEP!); road sides towards Lata Kijang, 3°11.42'N, 100°59.21'E, 459 m alt., 23 May 2019 (fr.), *Julius et al. FRI 73545* (KEP!).

##### Notes.

**Figure 2.**
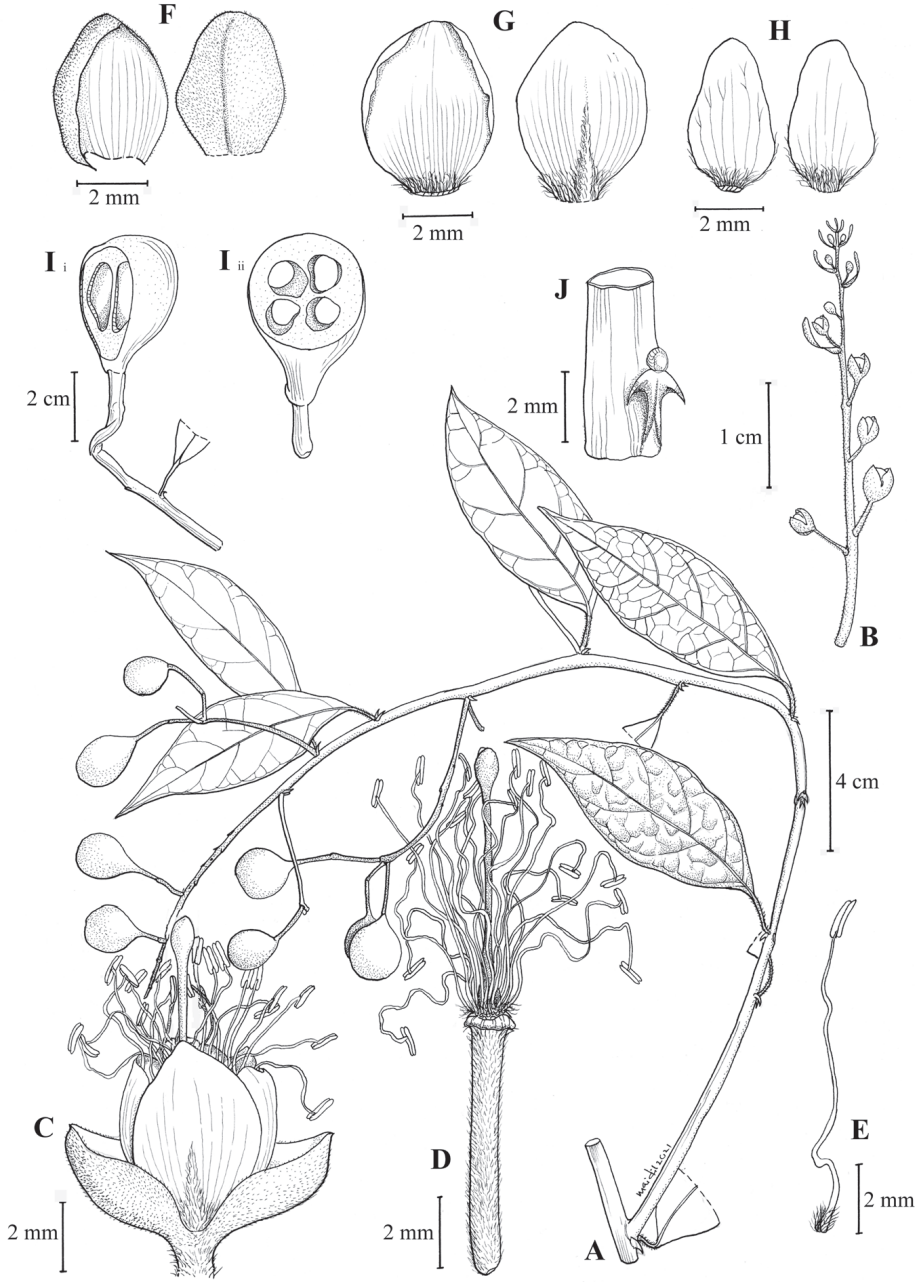
*Cappariskenaboiensis* Julius, sp. nov. **A** infructescence branch **B** inflorescence **C** flower **D** flower with sepals and petals removed exhibiting the stamens and ovary on gynophore **E** stamen **F** the outermost sepals pair **G** the dorsal petals **H** the ventral petals **I** the cross- and longitudinal-section of fruit **J** the stipular thorn close-up. (Drawn by Mohamad Aidil Noordin from *FRI57784*).

**Figure 3.**
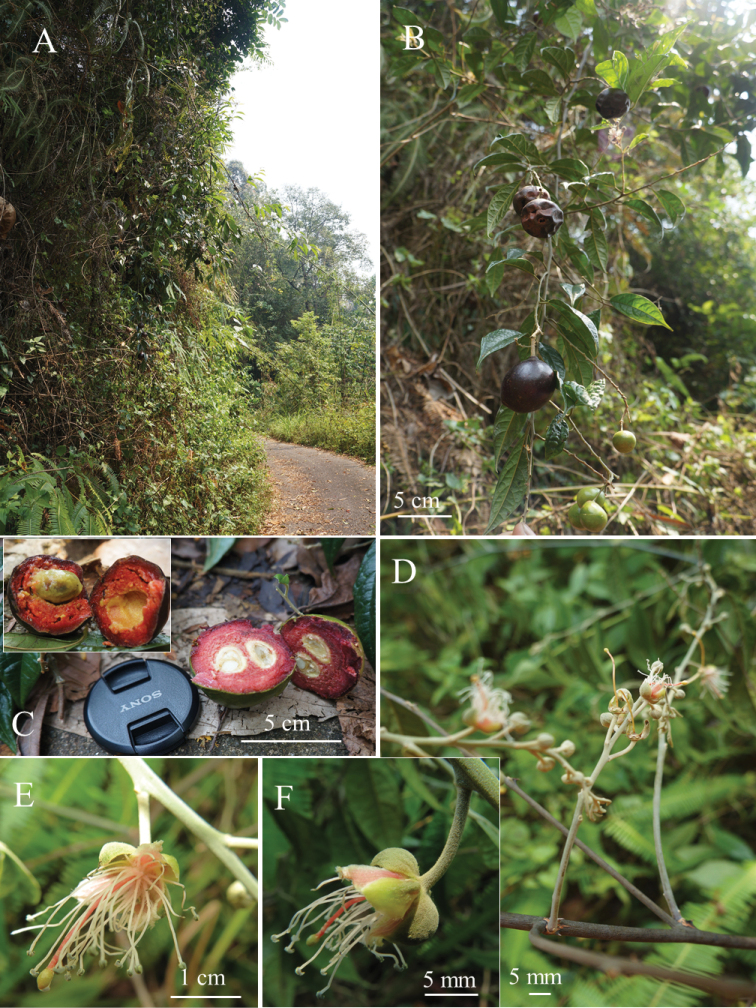
*Cappariskenaboiensis* Julius, sp. nov. **A** habitat **B** fruiting branch **C** cross-section of fruit (upper left, the cut ripe fruit) **D** inflorescence **E** flower, front view **F** flower, lateral-abaxial view. (Photographs **A–D** by A. Julius and **E–F** by K. Imin).

*Cappariskenaboiensis* (Figs 2, 3) is characterised by the flowers, loosely arranged in the raceme with each single flower subtended by a leaf-like bract. Vegetatively, *Cappariskenaboiensis* resembles *C.buwaldae* from the Seriales-group, but its short racemose inflorescences subtended by leaves are similar to members of the Subumbellates-Group. Characterised further by the small size of flowers with sepals 6–8 mm long and the plants hairy on young parts, *Cappariskenaboiensis* is best placed within the Subumbellates-Group (Jacobs 1965: 412). Further morphological comparison of this new species with other closely related species is as indicated in the Table 1.

**Table 1.** Morphological comparison of *Cappariskenaboiensis*, *C.buwaldae* and *C.longestipitata*.

Characters	Species		
	*C.kenaboiensis*	*C.buwaldae*	*C.longestipitata*
Leaf surface	Bullate	Bullate	Smooth
Leaf shape	Narrowly elliptic	Oblanceolate-elliptic	Oblong to slightly obovate
Leaf size	(5.5–)10–11.5 × (1.5–)3.5–5 cm	6–13(–23.5) × (1.5–)2.5–4.5(–8) cm	5–7 × 2.5–3.5 cm
Leaf base	Acute	Rounded to acute	Rounded
Leaf apex	Long acuminate to caudate, 1–1.5 cm long	Long acuminate to caudate, ca. 2 cm long	Acuminate, 0.4–0.7 cm long
Leaf hairs	Glabrous above, hairy beneath, denser on mid-rib and venation	Glabrous on both surfaces	Glabrous except for mid-rib beneath
Leaf colour when dried	Brownish-green	Brownish-green to yellowish-green	Olive green
Inflorescences	Simple racemose, axillary or terminal	In a series along the twig, 2–4, or supra-axillary	Compound racemose, Axillary or terminal
Bracts	Present	Absent	Absent
Sepals shape	Lanceolate, keeled	Ovate	Lanceolate, not keeled
Sepals size	6–8 mm long	3–5 mm long	3–5.5 mm long
Sepals hairs	Densely white hairs outside, glabrous inside	Glabrescent towards margin	Densely greyish puberulous outside
Fruit shape	Subglobose to pyriform	Shortly umbonate at the top and sometimes at the bottom	Unknown
Fruit exocarp	Smooth	Tuberculate	Unknown

The ripe fruits of this new species are similar to the edible fruits of *Capparisbuwaldea* - the smell is like a mangosteen, but the flesh is tasteless.

#### ﻿ Capparis scortechinii var. ruthiae

Taxon classificationPlantaeBrassicalesCapparaceae

Julius var. nov.

DF1941E5-E9C1-5D46-BBDD-74518FCB7AD2

urn:lsid:ipni.org:names:77254615-1

Figures 4 , 5

##### Diagnosis.

Recognised in the genus *Capparis* by the combination of the following characters: the racemes are strictly terminal and compact with flowers subtended by a conspicuous and persistent unguiculate, leaf-like bracts and the smaller [(13–)16–25 × 2–8 mm], greenish, thin leaf-like bracts covered with densely, tomentose, straw hairs on both surfaces soon glabrescent with age and the sepals (inner pair) glabrous along the margin and the filaments and gynophore both entirely glabrous and the petals loosely ciliate along margin.

##### Type.

Peninsular Malaysia. Pahang: Fraser’s Hill, 25 Jan 1982 (fl.), *Kiew RK3747* (holotype KLU!).

##### Description.

Climber ca. 1.5 m long or scrambling shrub (height unknown). ***Twigs*** straight covered with indumentum of dense, appressed, pale brown hairs soon glabrescent. ***Stipular thorns*** retrorse, tips 1–2 mm long. ***Leaves*** spirally arranged, but in one plane; petioles 10–15 × ca. 10 mm long, slender, young densely hairy with brown hairs soon glabrescent with age; lamina narrowly elliptic or oblong-elliptic, 8.5–11.5(–13.5) × 2.6–3.6 cm, chartaceous to subcoriaceous, drying brownish-green, base cuneate, margin flat and entire, apex acuminate and slightly caudate, with acumen 5–7(–10) mm long, glabrous above, sparsely hairy, denser on mid-rib beneath; mid-rib flat above, raised beneath; lateral veins 4–6 pairs, sunken above, distinct beneath, reddish when dry, looping towards the margin; intercostal veins reddish, finely reticulate, obscure above, distinct beneath. ***Inflorescences*** terminal, elongated racemes, rachis stout, straight, 3–8(–10) cm long, densely hairy with pale brown hairs; bracts leaf-like, unguiculate, lanceolate, (13–)16–25 × 2–8 mm, densely hairy with pale brown hairs on both surfaces. ***Flowers*** [in bud] many, compact, buds globose, 4–7 × 3–7 mm; pedicels 4–5 mm long, brownish; sepals 4, thin, green, orbicular to ovate, 4–6 × 4–6 mm, outer pair imbricate, densely hairy with appressed pale brown hairs outside, glabrous inside, inner pair with broadly transparent margin, glabrous on both surfaces; petals 4, pinkish, obovate, 6.5–7 × 4.5–5.5 mm, glabrous on both surfaces, except long, silky hairs at base of dorsal pair and dense, short silky hairs at base of ventral pair inside, loosely, ciliate along margin; stamens numerous, unequal length, filament pinkish, ca. 5–6 mm long, anther dorsifixed, ca. 1.5 × 0.5 mm; ovary ovoid, ca. 1.5 × 2.5 mm, on *gynophore* ca. 4 mm long, *glabrous*, stigma obscure. ***Fruits*** young green, mature fruit n.v., globose on stout gynophore, ca. 9.5 cm in diameter. ***Seeds*** many.

##### Vernacular names.

*Susoh beruga*, *kuku lang* (Malay).

##### Distribution.

**Figure 4.**
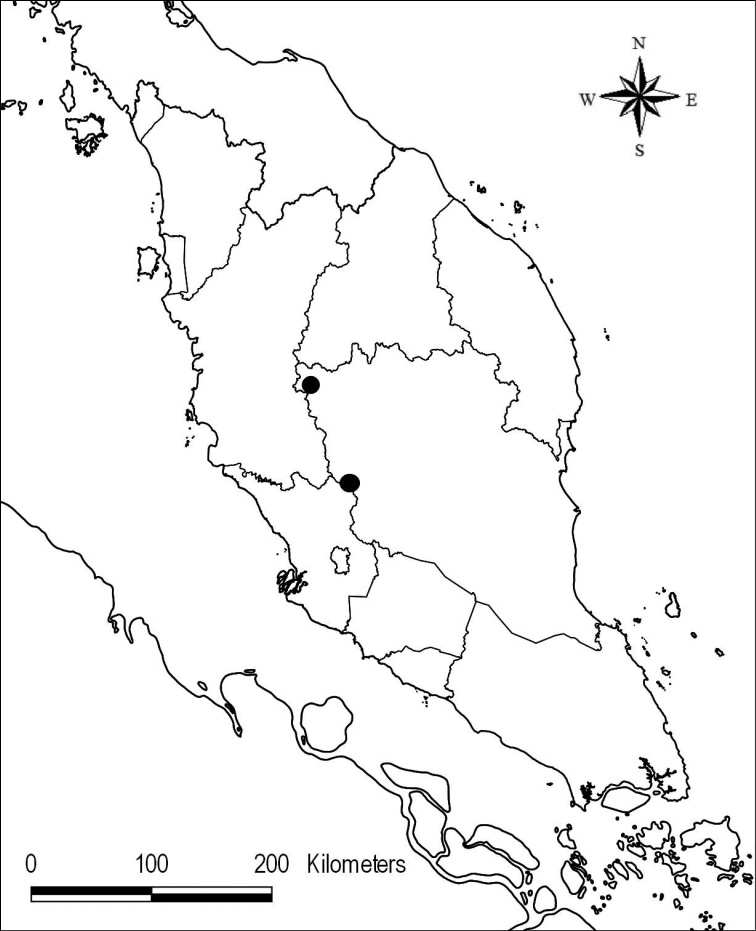
Distribution of Capparisscortechiniivar.ruthiae.

Endemic in Peninsular Malaysia, Pahang, known only from Fraser’s Hill and Cameron Highlands (Fig. 4).

##### Ecology.

On degraded hill slopes and open areas along the forest margins, gaps or roadsides, hill forest to lower montane forest at ca. 1280 m elevation.

##### Etymology.

This new variety is named after Dr Ruth Kiew, collector of type specimen and the Flora of Peninsular Malaysia project co-ordinator and consultant.

##### Conservation status.

Capparisscortechiniivar.ruthiae was conspicuous and inhabits forest margins, gaps or pathway near roadsides, but has not been relocated after 1993 at the original localities and adjacent areas even though they have been revisited many times. As the estimated extent of occurrence is less than 500 km^2^ and the declining of habitat quality, this variety is assessed as a Vulnerable B2ab(iii), following the IUCN Red List Categories and Criteria (IUCN 2001, 2012).

##### Notes.

**Figure 5.**
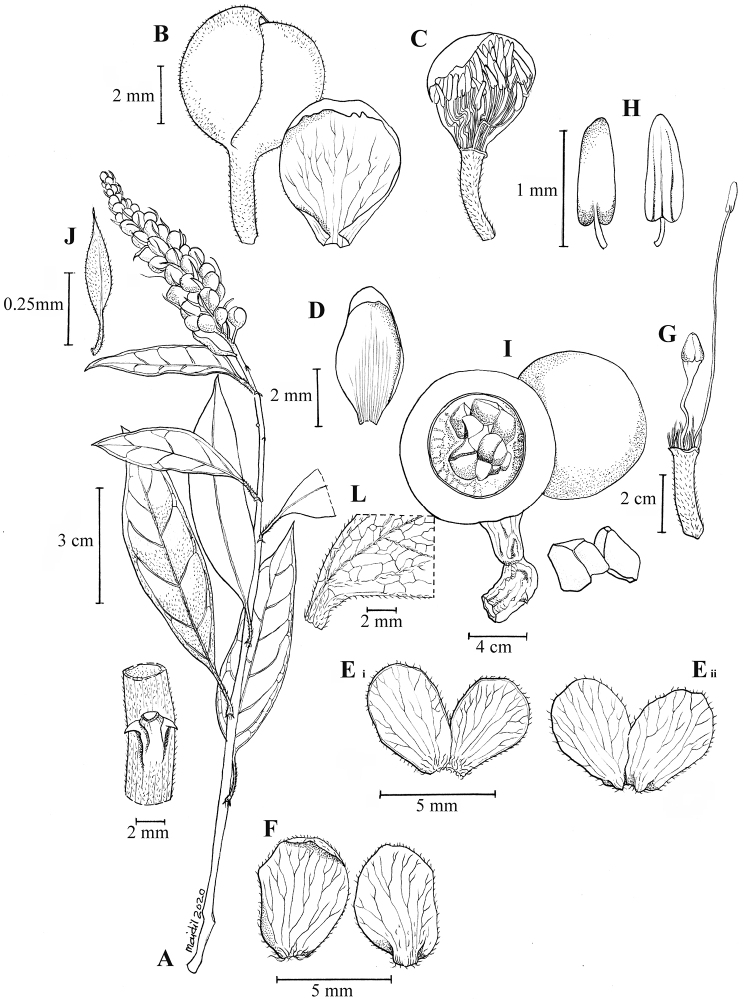
Capparisscortechiniivar.ruthiae Julius, var. nov. **A** flowering (bud) leafy twig with stipular thorns close-up next to it **B** flower bud close-up **C** flower bud with sepals and petals removed exhibiting the stamens **D** adaxial view of outer sepal (upper) and inner sepal (below) **E** dorsal petals, adaxial view (i) and abaxial view (ii) **F** ventral petals, exhibiting adaxial with hairs at base (left) and abaxial view (right) **G** flower bud dissected exhibiting one elongated stamen and the ovary on gynophore **H** anther, front (right) and back view (left) **I** fruit in cross-section and seeds (below) **J** an unguiculate leaf-like bract **K** venation close-up. (Drawn by Mohamad Aidil Noordin from Kiew *RK*3747 [**A–H, J, L**] and from Henderson *23551* [**I**]: the scale for **C** is similar to **B**).

Amongst the known members of Trinervia-group, Capparisscortechiniivar.ruthiae (Fig. 5) is closely related to var. scortechinii because both are characterised by the compact racemes. However, the new species has racemes strictly in terminal (but axillary rarely terminal in var. scortechinii) and the leaf-like bracts are smaller and greenish with tomentose, straw hairs on both surfaces, which is glabrescent with age abaxially (compared to a larger and prominent of velvety, shiny, rusty hairs in var. scortechinii). The new variety also differs in its entirely glabrous filament and gynophore (while var. scortechinii has the filament and gynophore densely hairy at the base), the petals are loosely ciliate along the margin (but glabrous in var. scortechinii), and the inner pair of sepals are glabrous along the margin (but ciliate in var. scortechinii).

Flowers with a vestigial and well-developed gynoecium are presented in Capparisscortechiniivar.scortechinii , but only well-developed gynoecium observed in var. ruthiae.

##### Additional specimens examined.

**Peninsular Malaysia. Pahang**: one-way road to Fraser’s Hill, 25 Jan 1982 (fl.), *Kiew RK1116* (KEP!); Fraser’s Hill, 1 Aug 1993 (fl.), *A. Zainuddin AZ4636* (KEP!); Fraser’s Hill, Jalan kecil to Gap, 19 Jun 1930 (fl.), *Kalong FMS22381* (KEP!); Fraser’s Hill, Richmond, 21 Apr 1955 (fr.), *Purseglove P4248* (L- image!, barcode L1856045) Cameron Highlands, 13 Apr 1930 (fr.), *Henderson SFN23551* (KEP!, SING!).

#### ﻿ Capparis trinervia var. chungiana

Taxon classificationPlantaeBrassicalesCapparaceae

Julius var. nov.

3C109AC5-5776-5752-B847-663FF9039F68

urn:lsid:ipni.org:names:77254616-1

Figures 6 , 7 , 8

##### Diagnosis.

Recognised in the genus *Capparis* by the combination of the following characters: the leaves broadly ovate-elliptic, relatively large (13–16 × 5.5–8.5 cm) and leaves drying reddish-brown, rarely pale green with pale yellow and rarely dark red venation, the tertiary venation obscure, the inflorescence strictly terminal with flowers arranged racemosely and becoming crowded at the distal part of the inflorescence, the stamens 30–40 and the fruit globose with ca. 11 cm in diameter.

##### Type.

Peninsular Malaysia. Selangor: Kuala Kubu Bharu to Fraser’s Hill Road, along the road side, 17 Apr 1971 (fl.), *Mahmud Sidek s.n.* (holotype KLU!).

##### Description.

Woody climber 9–12 m long. ***Twigs*** straight, covered with velvety, ferruginous hairs and glabrescent with age. ***Stipular thorns*** recurved downwards, tips 1.5–3 mm long. ***Leaves*** spirally arranged; petioles 15–20 × 2–3 mm, stout, incrassate or thickened, velvety hairy becoming glabrous; lamina broadly ovate sometimes ovate-elliptic, 13–16 × 5.5–8.5 cm, coriaceous to subcoriaceous, surface rugose and usually reddish-brown when dry, rarely pale green, base cuneate or occasionally rounded, margin flat and entire, apex acute or shortly cuspidate, with acumen ca. 4 mm long, glabrous on both surfaces; mid-rib flat above, raised beneath; lateral veins 5–7(–8) pairs, flat above, distinct beneath, pale yellow when dry, looping towards the margin; intercostal veins obscure. ***Inflorescences*** terminal, flowers arranged racemosely and becoming crowded at the distal part of the inflorescence rachis slender and straight; bracts caducous. ***Flowers*** [in bud] 4–7, buds globose, 1–1.8 × 1.3–1.8 cm; pedicels 3–3.5 cm long, swollen towards apex, velvety with ferruginous hairs; sepals 4, thick, whitish-green, orbicular, 1.2–1.5 × 1.2 cm, outer pair of sepals imbricate, covered with dense, velvety ferruginous hairs outside, glabrous inside, inner pair of sepals glabrous on both surfaces, except silky hairs at base outside; petals 4, cream or white with pink- or dark purple at base; stamens 30–40, unequal in length, anthers yellow; ovary green. ***Fruits*** young shiny and green, mature fruit n.v., globose to sub-globose on a stout gynophore, sometimes beaked when young, ca. 9 × 11 cm. ***Seeds*** (3–)12–15, sarcotesta whitish, testa thin and whitish-cream.

##### Vernacular names.

*Kuku lang* (Malay), *mentimun* (Temuan).

##### Distribution.

**Figure 6.**
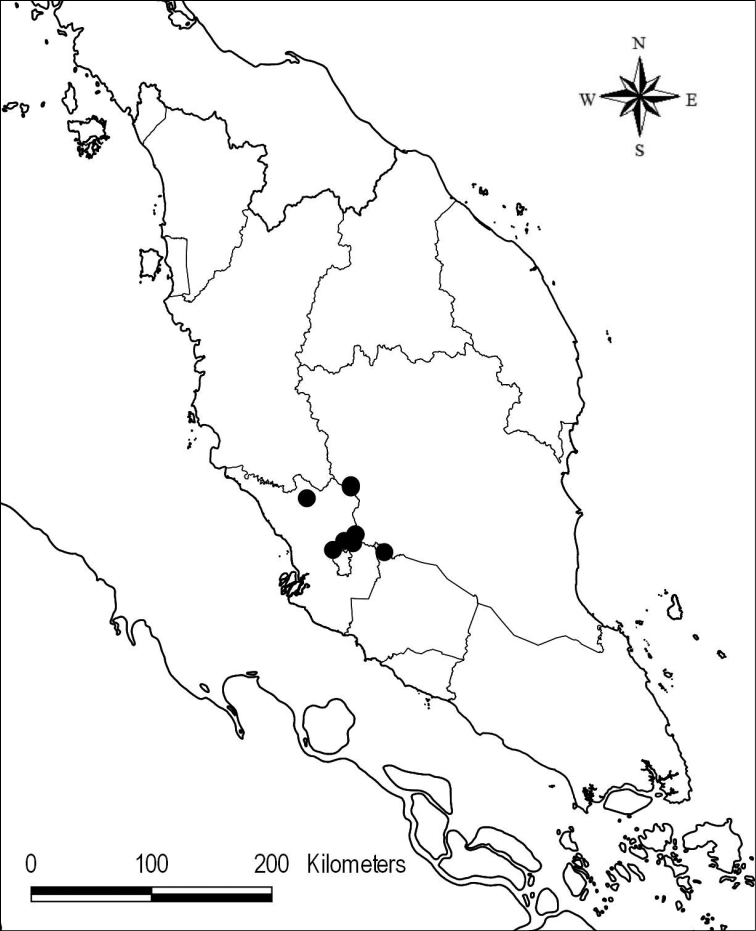
Distribution of Capparistrinerviavar.chungiana.

Endemic in Peninsular Malaysia, Negeri Sembilan, Selangor and Pahang (Fig. 6).

##### Ecology.

Lowland to lower montane forest, on forest edge or roadsides; 427–1067 m altitude.

##### Etymology.

The new variety is named after Dr Richard Chung Cheng Kong, Curator of the Kepong Herbarium (KEP) and Project Leader of Flora of Malaysia under 10^th^and 11^th^ Malaysian Plans.

##### Conservation status.

Capparistrinerviavar.chungiana inhabits forest margins, gaps or pathways near to roadsides, but is not a common species. This species has not been relocated in Selangor and Pahang even though the localities and adjacent areas have been revisited. This species, however, has been found and recorded from a new locality in Negeri Sembilan during a botanical survey in 2010, but it could not be relocated from two recent visits in 2014 and 2019. Recent field observations to Negeri Sembilan also show forest clearing pathways near the species habitat. Therefore, this species is assessed as Near Threatened following the IUCN Red List Categories and Criteria (IUCN 2001, 2012).

##### Additional specimens examined.

**Peninsular Malaysia. Selangor**: Ulu Gombak FR, 259 m (850 ft) alt., 19 Aug 1964 (fr.), *Mohd. Kasim & Mahmud Sidek 624* (KLU!); Gombak Forest, 16^th^ mile, followed path to stream and beyond, 427 m (1400 ft) alt., 12 Jun 1963 (fl.), *M.E.D. Poore 1189* (KLU!); Kepong Plantation Field 9J, hillslope, 10 Jan 1934 (fl.), *Abdul Hamid 33570* (KEP!); Ulu Langat, 30 Mar 1960 (fl., fr.), *Gadoh KL2072* (KEP! 2 sheets); Genting Sempah, 11 Dec 1970 (fl., fr.), *Kochummen FRI16263* (KEP!). **Pahang**: Fraser’s Hill, near gate at gap, roadside, 1067 m (3500 ft) alt., 28 Feb 1979 (fl., fr.), *Kochummen FRI18471* (KEP!); road to Fraser’s Hill, climber on Mahang tree beside the road, 914 m (3000 ft) alt., 26 Aug 1981 (fr.), *K.M. Wong FRI32242* (KEP!). **Negeri Sembilan**: Jelebu, Kenaboi FR, G. Besar Hantu, near Kg. London area, 3°11.00'N, 102°00.76'E, 500 m alt., 6 May 2010 (fr.), *Julius et al. FRI64006* (KEP!).

##### Notes.

**Figure 7.**
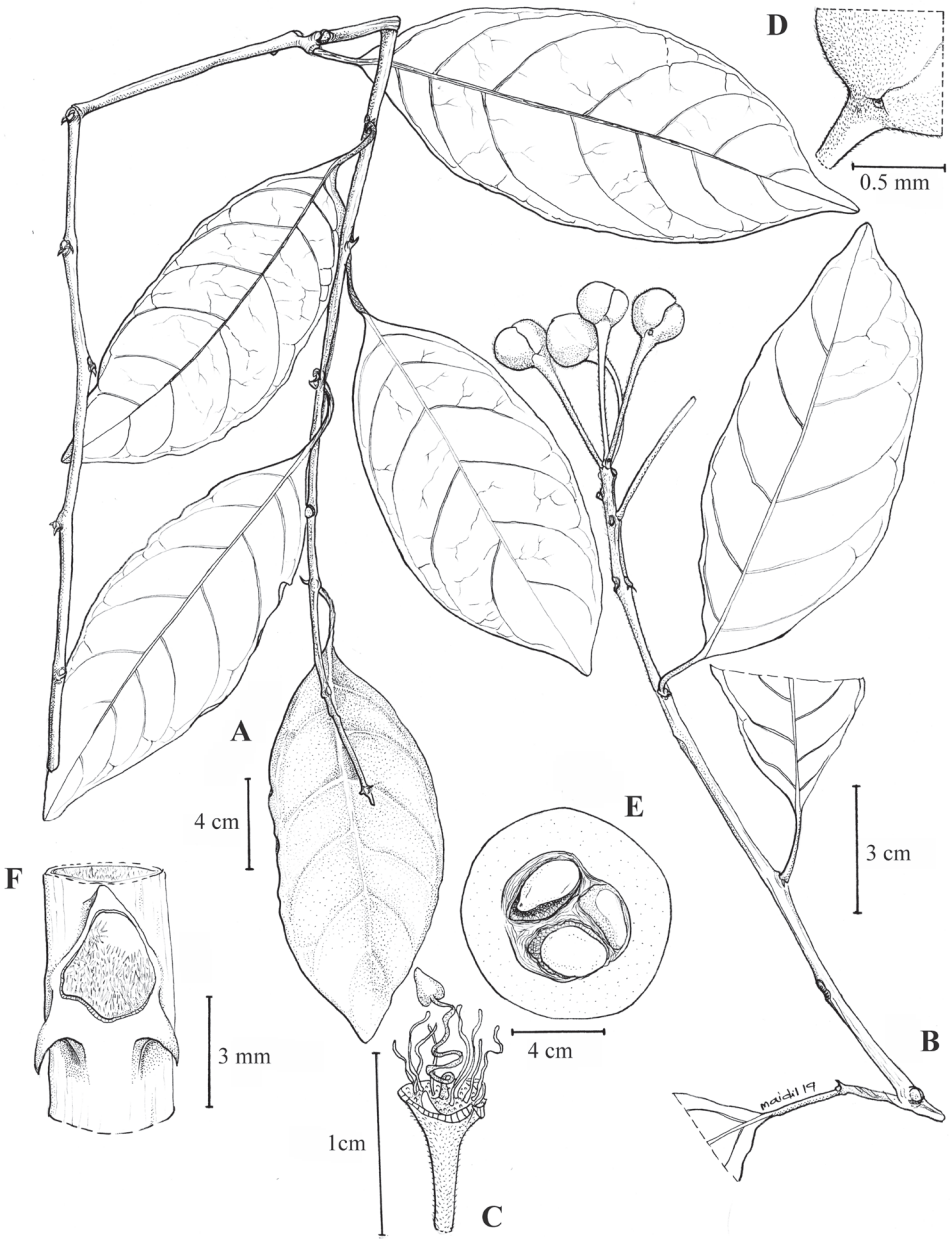
Capparistrinerviavar.chungiana Julius, var. nov. **A** the leafy branch **B** the flowering (bud) leafy twig **C** flower with sepals and petals removed exhibiting the filaments and ovary on gynophore **D** upper pedicel and base of calyx **E** the fruit in cross section **F** stipules close-up. (Drawn by Mohamad Aidil Noordin from *FRI64006* [**A, E, F**] and Mahmud Sidek s.n. [**B–D**]).

**Figure 8.**
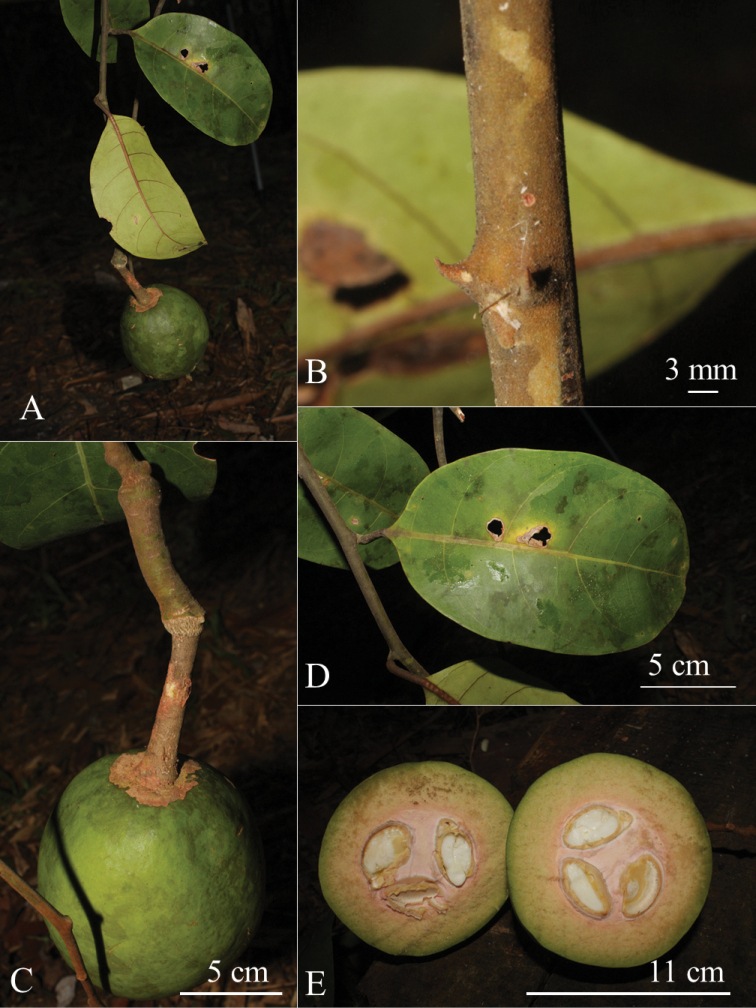
Capparistrinerviavar.chungiana Julius, var. nov. **A** fruiting twig **B** stipular thorns **C** young fruit on stout gynophore **D** leaf **E** young fruit, cross section. (Photographs by K. Imin).

The comparatively large flowers, with sepals up to 1.5 cm long, place Capparistrinerviavar.chungiana (Figs 7, 8) in the Trinervia-Group (Jacobs 1965; Srisanga and Chayamarit 2004). Specimens of this new species were determined as *C.erycibe*, no doubt due to the comparatively large leaves, but it differs in having the flowers arranged racemosely and becoming crowded at the distal part of the inflorescence rather than the paniculate arrangement in *C.erycibe*. In addition, *C.erycibe* has smaller flowers with sepals only 4–6 mm long and was placed within the Subumbellates-group (Jacobs 1965: 411).

The flower colour of this species has been described as ‘white’ or ‘cream’ with pink or dark purple at base in the label notes. Unfortunately, the open flowers are missing from the specimen sheets. Additionally, there are no new flowering specimens from the recent collections and full details of the flowers, such as measurement of the mature flowers and bracts, remain incomplete. Moreover, the dissected parts of the rehydrated flower buds are in poor condition and mostly do not contain either androecium or gynoecium, the cause of which is unknown.

However, this new variety still can be distinguished from other closely related members of the Trinervia-Group (see Table 2 for the comparison details) or species known from Peninsular Malaysia vegetatively. For example, the relatively large fruit (ca. 11 cm in diam.) of Capparistrinerviavar.chungiana is similar to *C.scortechinii* (ca. 11 cm in diam.) and *C.trisonthiae* from Thailand (6–8 cm in diam.), but the latter two differ in their densely racemose and paniculate inflorescences, respectively; and the flowers arranged racemosely and becoming crowded at the distal part of the inflorescence, though has similarity to typical var. trinervia, but var. chungiana differs by its subcoriaceous to coriaceous leaves with broadly ovate to ovate-elliptic lamina (compared to chartaceous with oblong-elliptic or broadly lanceolate lamina in var. trinervia), the drying leaves are reddish-brown, rarely pale green with venation pale yellow, rarely dark red on both surfaces (compared to dull green with brownish main nerves on both surfaces in var. trinervia), the intercostal veins are obscure (compared to irregular reticulate and distinct in var. trinervia) and the stamens are 30–40 (whereas in var. trinervia, (30–)60–70).

**Table 2.** Morphological comparison of Capparistrinerviavar.chungiana, C.trinerviavar.trinervia and *C.trisonthiae*.

Characters	Species		
	C.trinerviavar.chungiana	C.trinerviavar.trinervia	*C.trisonthiae*
Stipular thorns	1.5–3 mm long, recurved downwards	1–3(–6) mm long, patent or recurved, mostly downwards, rarely upwards	2–4 mm long, recurved downwards
Petiole	1.5–2 cm long, 2–3 mm in diam., stout	0.7–1.8 cm long, ca. 1.5 mm in diam., slender	ca. 2 cm long, ca. 4 mm in diam., stout
Leaf shape	Broadly ovate to ovate-elliptic	Oblong-elliptic or broadly lanceolate	Broadly ovate
Leaf size	13–16 × 5.5–8.5 cm	(5–)10–14(–19) × (2–)3.5–8.5 cm	(10–)12–18 × (6.5–)8–11 cm
Leaf apex	Acute or shortly cuspidate	Obtuse to shortly acuminate	Retuse to emarginate
Leaf hairs	Glabrous on both surfaces	Glabrescent	Glabrous on both surfaces
Leaf colour when dried	Reddish-brown, rarely pale green with pale rarely dark red venation on both surfaces	Dull green with brownish venation on both surfaces	Dull green above, brown beneath
Intercostal veins	Obscure on both surfaces	Irregular reticulate, distinct on both surfaces	Irregular reticulate, prominent on both surfaces
Inflorescences	Racemes with flowers becoming crowded at the distal part of the inflorescence	Racemes with flowers becoming crowded at the distal part of the inflorescence or subumbels	A leafy paniculate
Sepals shape	Orbicular	Orbicular	Lanceolate
Sepals size	1.2–1.5 cm long	0.9–1.5 cm long	1.8–2.5 cm long
Sepals hairs	Densely velvety ferruginous hairs abaxially, glabrous adaxially	Densely orange-yellow puberulous abaxially, glabrous adaxially	Ferruginous puberulous abaxially, puberulous adaxially in the marginal parts
Stamens	30–40	(30–)60–70	140–170
Fruit	Globose, ca. 11 cm in diam.	Globose, 3.5–5 cm in diam.	Ellipsoid, 6–8 cm in diam.

#### Checklist of *Capparis* species in Peninsular Malaysia

(Bt. = Bukit (hill); FR (Forest Reserve); G. = Gunung (mountain); P. = Pulau (islands); WR (Wildlife Reserve))

**1. *Capparisacutifolia* Sweet** Hort. Brit. 2^nd^ ed. (1830) 585; Jacobs, Blumea 12, 3 (1965) 426, p.p. excluding subsp. bodinieri (H.Lév.) Jacobs, subsp. obovata Jacobs and subsp. sabiaefolia (Hook.f. & Thoms.) Jacobs and subsp. viminea (Hook.f. & Thoms.) Jacobs; Chayamarit, Fl. Thailand 5, 3 (1991) 249.

**Type.** Myanmar [Burma], Kurz 1826 (holotype CAL-photo! barcode CAL0000004987; isotype K!, barcode K000247293).

**Distribution.** In Peninsular Malaysia, so far collected from Kedah (Gua Labua).

**Ecology.** Grows on rock crevices in the limestone forest at low altitude ca. 191 m.

**Conservation status.** This species is rare. It was recently collected in 2008 in a forest area. It is assessed as Endangered B2ab(iii) because the small area of occupancy (< 500 km^2^), known only from a single locality.

**Specimens examined. Peninsular Malaysia. Kedah**: Sik, Ulu Muda FR, Gua Labua, limestone hill, 3 Mar 2008, *Rafidah et*. *al. FRI 55677* (KEP!).

**Notes.***Capparisacutifolia* is characterised by the absent of stipular thorns, the inflorescence with 1–2(–5)-flowers in a series along the twig and the extra membranous lamina is ovate with acuminate-caudate apex. There are five subspecies treated under this species (Jacobs 1965), but the other four have all been elevated to species rank: subsp. sabiaefolia (Hook.f. & Thoms.) Jacobs and subsp. obovata Jacobs as *Capparissabiifolia* Hook.f. & Thoms. (Chayamarit 1991), subsp. bodinieri (H.Lév.) Jacobs as C.bodinieri H.Lév. and subsp. viminea (Hook.f. & Thoms.) Jacobs as *C.membranifolia* Kurz (Zhang and Tucker 2008; Maurya et al. 2020)

**2. *Cappariscucurbitina* King** J. As. Soc. Beng. 58, 2 (1889) 395; Jacobs, Fl. Malesiana 1, 6 (1960) 85, Blumea 12, 3 (1965) 446.

**Type.** Peninsular Malaysia, Perak, Ulu Bubu, *King’s Coll. 10027* (lectotype designated by Jacobs; CAL-photo!, barcode CAL0000005012, CAL0000005013, CAL0000005014 & CAL0000005015; isolectotypes G, barcode G00237951, K!, barcode K000643986)

**Distribution.** Endemic in Peninsular Malaysia and restricted to Perak with few collections from Ulu Bubu (*King’s Coll. 8824* & *King’s Coll. 10795*, CAL-photo!), Dindings (*Ridley 10261*, SING!), Parit Forest Reserve (*Symington FMS39490*, SING!) and the type.

**Ecology.** In dense forest at 152–244 m altitude.

**Conservation status.** This species is very rare since it has been collected only four times, the last time being in 1899. Despite several visits made more recently to the localities mentioned above, *Cappariscucurbitana* could not be located. As the estimated extent of occurrence is less than 5000 km^2^ and the quality of habitat is declining, *C.cucurbitina* is assessed as Endangered B1ab(iii), according to the IUCN Red List Categories and Criteria (IUCN 2001, 2012)

**Specimen examined. Peninsular Malaysia. Perak**: Manjung, Dinding, 1899, *Ridley 10261* (SING!); Kinta, Parit FR, 8 Apr 1935, *Symington FMS39490* (SING!).

**Notes.** Amongst the species with inflorescence in a series along the stem, *Cappariscucurbitina* can be easily identified by its torulose fruits.

**3. *Capparisdiffusa* Ridl.** J. Str. Br. R. As. Soc. 59 (1911) 68; Jacobs, Fl. Malesiana 1, 6 (1960) 81, Blumea 12, 3 (1965) 447, figs 3, 26; Chayamarit, Fl. Thailand 5, 3 (1991) 254.

**Type.** Peninsular Malaysia, Perlis, Bt. Lagi, *Ridley 15174* (holotype SING! barcode 0056837; isotype K! barcode K000643988).

**Distribution.** In Peninsular Malaysia, this species found in Perlis, Kedah (Langkawi Islands), Perak (G. Pondok) and Pahang (Bt. Serdam, Gua Kechil).

**Ecology.** Evergreen forest on limestone rocks and quite common on dry rocky hill slopes.

**Conservation status.** There are several voucher specimens which have been collected as recently as 2008. None of the habitats is within protected areas. As the estimated extent of occurrence is less than 20,000 km^2^ and the quality of habitat is declining, *Capparisdiffusa* is assessed as Vulnerable B1ab(iii), according to the IUCN Red List Categories and Criteria (IUCN 2001, 2012).

**Specimens examined. Peninsular Malaysia. Kedah**: Langkawi, P. Jerkom, 6°26.67'N, 99°78.33'E, *Stone* 6988 (KLU!). **Perak**: Kuala Kangsar, G. Pondok, Padang Rengas, 4°77.63'N, 100°83.70'E, *Chin 870* (KLU!). **Pahang**: Raub, Bt. Serdam, limestone, near the summit, ground rocky with little soil, vegetation fairly dense scrubby, 3°83.33'N, 101°91.67'E, *Chin 1104* (KLU!); Gua Kechil, 3°83.71'N, 101°93.37'E, *Julius FRI 56286* (KEP!).

**Notes.** Amongst the species with small leaves, *Capparisdiffusa* is distinguished by its terminal or lateral inflorescences on short twigs and the 3–5-flowered in umbellate arrangement, with 1–2 small leaves, sometimes a few umbels united to a small panicle and sessile.

**4. *Cappariskenaboiensis* Julius**

**5. *Cappariserycibe* Hallier f.** Bull. Herb. Bossier 6 (1898) 216; Jacobs, Fl. Malesiana 1, 6 (1960) 75, fig. 8, Blumea 12, 3 (1965) 449; Chayamarit, Fl. Thailand 5, 3 (1991) 251.

**Type.** Indonesia, Java, Jambi [Tjampea], *Hallier f*. 779A (holotype BO-photo!, acc. no. 134004-134005; isotype L-photo! barcode L0035324). *Synonym: Capparis paniculata* Ridl., J. Fed. Malay St. Mus. 10, 2 (1920) 129. *Type*: Peninsular Malaysia, Kelantan, near Chaning Estate, Kelantan River, Feb 1917, *Ridley s.n.* (holotype SING!, barcode 0096891]; isotype K!, barcode K000247300).

**Distribution.** In Peninsular Malaysia, this species is collected from Kelantan (Chaning Woods) and Pahang (Kuala Tembeling).

**Conservation status.** This species is very rare since it has been collected only twice, the last time being in 1917. Although a fieldwork expedition has been made to the localities, *Cappariserycibe* could not be located. As the estimated of extent occurrence is less than 100 km^2^ and the declining of habitat quality, in which the type locality was converted into an oil palm estate, the species could be assessed as Critically Endangered, according to the IUCN Red List Categories and Criteria (IUCN 2001, 2012). However, the species has also been collected from a National Park, which is a protected area. Therefore, this species is assessed as Near Threatened.

**Ecology.** In the lowland tropical forests.

**Specimens examined. Peninsular Malaysia. Pahang**: Jerantut, Tembeling, Kuala Tembeling, Aug 1891, *Ridley s.n*. (SING).

**Notes**. This species easily recognised by its paniculate inflorescences.

**6. CapparismicracanthaDC.subsp.micracantha** Prod. 1 (1824) 247; Jacobs, Fl. Malesiana 1, 6 (1960) 85, Blumea 12 (1965) 466; Chayamarit, Fl. Thailand 5, 3 (1991) 246.

**Type.** Indonesia, Java, *Lahaye s.n.* (holotype G-DC! barcode G00203273).

**Vernacular names.***Jambul merak*, *melada* (Malay).

**Distribution.** In Peninsular Malaysia, it is occurring in Perlis (Kangar, Bt. Papan FR), Penang (P. Badak, P. Tikus), Kedah (Alor Setar, Langkawi), Kelantan (Kota Bharu) and Terengganu (Kuala Terengganu).

**Conservation status.** This species is widely distributed in the northern part of Peninsular Malaysia and is assessed as Least Concern.

**Ecology.** On limestone and sandy spots, or riverside at low altitude below 500 m.

**Specimens examined. Peninsular Malaysia. Perlis**: Kangar, 13 Jul 1936, *Corner SFN31557* (KEP!), *Ridley s.n.* (KEP!). **Kedah**: Alor Setar, *Haniff SFN10449* (SING!), *Haniff 10470* (SING!), *Ridley 15175* (SING!); Langkawi, P. Batang, *Rosdi et al. FRI59928* (KEP!); Sept 1900, *Haniff s.n.* (SING!); P. Pasir, 2 Mar 1982, *Stone 15032* (KEP!); P. Selang, *Corner s.n.* (SING!); P. Selang, Tanjung Botol, 24 Feb 1970, *Stone 9084* (KLU!); P. Singa Besar, 24 Feb 1982, *T. & P. 718* (KLU!); P. Timun, 24 Nov 1934, *Henderson SFN29124* (KEP!). **Penang**: Badak Mati, near the beach, *Curtis 1762* (SING!); P. Tikus, Mac 1889, *Curtis 1762* (SING!). **Kelantan**: Kota Bharu, *Corner s.n.* (KEP!). **Terengganu**: Kuala Terengganu, *Holttum 15190* (KEP!); Kg. Padang Negara, *Sinclair 39811* (KEP!).

**Notes.**Capparismicracanthasubsp.micracantha has smaller flowers compared to Capparismicracanthasubsp.korthalsiana. In addition, it has less than 20 stamens, whereas in subsp. *Korthalsiana*, the stamens are numerous.

**7. CapparismicracanthaDC.subsp.korthalsiana (Miq.) Jacobs** Fl. Malesiana 1, 6 (1960) 86. *Synonyms: Capparis korthalsia* Miq., Illustr. (1870) t.17.

**Type.** Borneo, S Kalimantan, Pulu Lampei, *Korthals s.n.* (holotype L-photo! barcode U0000960); *C.finlaysoniana* Wall. ex Hook. f., Fl. Brit. India 1 (1875) 179. *Type*: Singapore, *Wallich 6992B* (holotype K-W!, barcode K000643985).

**Distribution.**Capparismicracanthasubsp.korthalsiana is distributed in the southern part of Peninsular Malaysia and it has been collected from Pahang (Rompin) and Johor (Mersing and Masai).

**Conservation status.**Capparismicracanthasubsp.korthalsiana has been found and collected only four times, the last time being in 1967. With small distribution, the extent of occurrence less than 5000 km^2^ and the declining of habitat, C.micracanthasubsp.korthalsiana could be assessed as Endangered, according to the IUCN Red List Categories and Criteria (IUCN 2001, 2012) . However, C.micracanthasubsp.korthalsiana was also found from Pulau Aur, which is a protected habitat under the Marine Park Act. Thus, C.micracanthasubsp.korthalsiana is assessed as Near threatened.

**Ecology.** Its habitat preference is for wetter forest than that preferred by C.micracanthasubsp.micracantha.

**Specimens examined. Peninsular Malaysia. Pahang**: Rompin, Sg. Aur, SE Pahang Aur FR at Sg. Aur, 11 May 1967, *Whitmore FRI 3689* (KEP!), 20 Apr 1919, *Yeop 3178* (KEP!). **Johor**: Mersing, P. Aur, Oct 1892, *Fielding s.n.* (SING!); Masai, Hook Linn Estate, 6 Mar 1948, *Mc Caul 38407* (KEP!).

**Notes.***Capparismicracantha* subsp. *korthalsiana* differs from C.micracanthasubsp.micracantha in several aspects, but easily distinguished by its inflorescences being supra-axillary, either solitary or in pairs and the stamens are numerous.

**8. *Capparispubiflora* DC.** Prod. 1 (1824) 246; Jacobs, Fl. Malesiana 1, 6 (1960) 82, figs 16, 17, Blumea 12 (1965) 479; Chayamarit, Fl. Thailand 5, 3 (1991) 258.

**Type.** Indonesia, Timor, *s.c.*, (holotype P-photo! MNHN-P-P05461890; isotype G-DC! barcode G00207275). *Synonyms: Capparispubiflora* var. perakensis Scort. ex King, J. As. Soc. Beng. 58, 2 (1889) 394, *C.perakensis* (Scort. ex King) Ridl., Fl. Malay Pen. 1 (1922) 124. *Type*: Peninsular Malaysia, Perak, Kuala Dipang FR, *Scortechini 1784* (holotype CAL n.v.; isotype K!, barcode K000643987).

**Distribution.** In Peninsular Malaysia, it has been found so far from Perak, Kelantan (Gua Musang), Pahang (G. Senyum, Gua Kechil, Bt. Chintamanis and Bt. Cheras) and Selangor (Batu Caves).

**Conservation status.***Capparispubiflora* is found and collected mostly from limestone hills with a recent collection from Gua Musang (*FRI94559*). None of the localities is within protected areas and some limestone hills are active quarries. Therefore, this species is assessed as Near Threatened.

**Ecology.** Straggling on floor of a crack in limestone cliff (*Henderson SFN 22319*, *Wan Syafiq FRI94559*), also in damp shady forest (*Scortechini 1784*).

**Specimens examined. Peninsular Malaysia. Kelantan**: Gua Musang, Felda Perasu, Ktn 84, limestone hill, 26 Sept 2019, *Wan Syafiq FRI94559* (KEP!). PAHANG: Kuantan, Bt. Cheras, 10 Oct 1931, *Henderson SFN25220* (KEP!); Temerloh, G. Senyum, on floor of cleft in limestone clift, 31 Jul 1929, *Henderson SFN22319* (KEP!); Bentung, Bt. Chintamani, 31 Jul 2009, *Julius FRI 56292* (KEP!); Raub, Gua Kechil, 30 Jul 2009, *Julius FRI56289* (KEP!). **Perak**: Kinta, Kuala Dipang FR, *Scortechini 1784* (K!); Kuala Kangsar, Sg. Siput, quarried hills near Jalong, limestone hill, 29 Jan 2018, *Rafidah et al. FRI82018* (KEP!). **Selangor**: Gombak, Batu Caves, 14 Sept 1920, *Burkill SFN6369* (KEP!).

**Notes.***Capparispubiflora* easily recognised by its comparatively large oblanceolate leaves, 13.5–21 × 4–8.5 cm and the axillary fasciculate inflorescences.

**9. Capparisscortechiniivar.scortechinii King** J. As. Soc. Beng. 58, 2 (1889) 394; Jacobs, Fl. Malesiana 1, 6 (1960) 73, Blumea 12 (1965) 488.

**Type.** Peninsular Malaysia, Perak, Batang Padang District, *King’s Coll. 8083* (holotype CAL; isotype K! barcode K000643959).

**Vernacular names.***Kuku lang* (Malay), *akar pengsek*, *mentimun* (Temuan).

**Distribution.** In Peninsular Malaysia: Penang (Batu Feringgi), Perak (Piah FR), Terengganu (Ulu Brang), Pahang (Taman Negara, G. Benom) and Selangor (Bt. Ranggong, Bt. Putih FR).

**Conservation status**. Capparisscortechiniivar.scortechinii has been found and collected from at least five states in Peninsular Malaysia, which is considered widely distributed and could be assessed as Least Concern. However, the population has been found and collected mostly from unprotected areas and, thus, this species is assessed as Near Threatened.

**Ecology.** In rain forest, lowland areas.

**Specimens examined. Peninsular Malaysia. Penang**: Batu Feringgi, May 1885, *Curtis 239* (SING!). **Perak**: Hulu Perak, Piah FR, 16 Jul 1967, *Kochummen FRI2464* (KEP!); Batang Padang, *King’s collector 8083* (K!). **Terengganu**: Hulu Terengganu, Sekayu, Bt. Lanjut FR, 20 Sept 1969, *Shing FRI13503* (KEP!); Ulu Berang, Jul 1937, *Moysey SFN33676* (KEP!). **Pahang**: Temerluh, Krau WR, G. Benom, Ulu Chelon, 24 Mar 1967, *Whitmore FRI3434* (KEP!), logging track from Ulu Cheka to G. Benom, 10 Jan 1978, *Ng FRI27160* (KEP!); Jerantut, Taman Negara, Bt. Terom, near Kuala Keniyum, 5 Mar 1968, *Whitmore FRI8550* (KEP!); Bentung, Ulu Langat FR, Sempadan Looi, 30 Mar 1960, *Gadoh KL2072* (KEP!). **Selangor**: Petaling, Bt. Cherakah FR, 18 Jul 1986, *FRI33522* (KEP!); Hulu Selangor, Kuala Kubu - Raub Road, along the road side, *Mahmud* s.n. (KEP!); Hulu Langat, Bt. Rangong, Kuala Looi, 13 Mar 1960, *Gadoh KL2044* (KEP!); Hulu Langat, Kuala Pansom, 10 Sept 1958, *Gadoh KL 862* (KEP!); Gombak, Gombak FR, Mile 16, followed path to stream and beyond, 2 Jun 1963, *Poore 1189* (KEP!); ibid., Ulu Gombak FR, edges, 19 Aug 1964, *Mohd. Kasim 625* (KEP!); ibid., Bukit Putih FR, Bkt. Sg. Puteh, 6 Feb 1926, *Mat Yassin 10837* (KEP!); ibid., Kepong Plantation, Field 9J, 10 Feb 1934, *Abdul Hamid FMS33570* (KEP!); ibid., Kepong Plantation, Field 5, 10 Feb 1934, *Abdul Hamid FMS36005* (KEP!).

**Notes.**Jacobs (1965), in his description of this species, mentions that the gynophore is glabrous, but actually densely hairy at the basal part. The filament is also densely hairy at base. This observation has been confirmed by examining few specimens including the one (*Curtis 239*, SING) cited in his treatment.

Though the inflorescence has been described in the terminal position by Jacobs (1965), but observed only in *King’s Coll. 8083* (K), other specimens examined have axillary inflorescences, except in *Curtis 239*, where both axillary and terminal inflorescences occur. Thus, axillary inflorescences are more common in Capparisscortechiniivar.scortechinii.

Two specimens from Fraser’s Hill (*Kalong 22381*, *Pursglove P4248*), cited in Jacobs (1965), have terminal inflorescences, but these belong to Capparisscortechiniivar.ruthiae.

**10. Capparisscortechiniivar.ruthiae** Julius

**11. *Capparissepiaria* L.** Syst. Nat., ed. 10. 2: 1071 (1759); Jacobs, Fl. Malesiana 1, 6 (1960) 79, Blumea 12 (1965) 489; Chayamarit, Fl. Thailand 5, 3 (1991) 241.

**Type.** India, *Linnean. Cat. 664.4* (holotype LINN-photo!).

**Distribution**. In Peninsular Malaysia, so far collected from Kedah (Alor Setar and Kg. Nangka) and Kelantan.

**Conservation status**. This species is very rare with only four specimens recorded from Kedah and Kelantan and it was last collected in 1933. As the estimated extent of occurrence is less than 5000 km^2^ and the declining of habitat quality, *Capparissepiaria* is assessed as Endangered B1ab(iii), according to the IUCN Red List Categories and Criteria (IUCN 2001, 2012) .

**Ecology.** In lowland forest.

**Specimens examined. Peninsular Malaysia. Kelantan**: Gua Musang, Batu Neng, limestone hill, 200–300 m alt., near the summit, on dry rocky terrain, *Chin 1542* (KLU!); (locality unknown), Mac 1881, *King’s collector 1415* (SING!). **Kedah**: Kampung Nangka, *Holttum 19827* (SING!); Alor Setar, *Mohamed Nur 15176* (SING!).

**Notes.** Amongst the species with small leaves in this genus, *Capparissepiaria* is easily recognised by its conspicuously flexuose twigs.

**12. *Capparistrinervia* Hook.f. & Thoms.** Fl. Brit. India 1 (1875) 175; Jacobs, Fl. Malesiana 1, 6 (1960) 73, Blumea 12 (1965) 500. *Synonym: Capparis kuntslerii* King, J. As. Soc. Beng. 58, 2 (1889) 396; Ridley, Fl. Malay Pen. 1 (1922) 122.

**Type.** Peninsular Malaysia, Perak, G. Bubu, *King’s Coll. 8337* (holotype CAL-photo!, barcode CAL0000004992, CAL0000004993, CAL0000005007; isotype K!, barcode K000643958).

**Distribution**. In Peninsular Malaysia so far collected from Kedah (Alor Setar) and Kelantan. Recent surveys in 2010 were unable to locate the species.

**Conservation status**. Data Deficient.

**Ecology.** Found in hill forest at low altitude, 122–244 m.

**Notes.** This species is best distinguished from others by combination of several characters: the leaves are trinerved at base and the irregular reticulate venation distinct on both surfaces and the inflorescences are terminal with flowers arranged in subumbels.

**13. Capparistrinerviavar.chungiana Julius**

**14. *Capparisversicolor* Griff.** Not. Pl. As. (1854) 577; Jacobs, Blumea 12 (1965) 501, fig. 32; Chayamarit, Fl. Thailand 5, 3 (1991) 256.

**Type.** Myanmar [Burma *Griffith 936*] (holotype K!, barcodes K000247350, K000247351). *Synonym: Capparis larutensis* King, J. As. Soc. Beng. 58 (1889) 393, Jacobs, Fl. Malesiana 1, 6 (1960) 89. *Type*: Peninsular Malaysia, Perak, Larut, *King’s Coll. 5103* (holotype CAL-photo!, barcodes CAL0000004975–CAL0000004979; isotype K!, barcode K000643984).

**Distribution**. In Peninsular Malaysia, recorded so far from Perak (Larut and Kampar).

**Conservation status**. This species is very rare with only two specimens recorded from Perak and it was last collected in 1898. As the estimated extent of occurrence is less than 5000 km^2^ and the declining of habitat quality, *Capparisversicolor* is assessed as Endangered B1ab(iii), according to the IUCN Red List Categories and Criteria (IUCN 2001, 2012).

**Ecology.** In thickets or dense forest, clinging on trees, at altitude below 200 m.

**Specimen examined**. Peninsular Malaysia. Perak: Kinta, Kampar, Sept 1898, *Ridley 9646* (SING!).

**Notes.***Capparisversicolor* is amongst the species having relatively small leaves less than 5 cm long. It has a simple corymb inflorescence terminally on a short lateral twig with few leaves below.

## Supplementary Material

XML Treatment for Capparis kenaboiensis

XML Treatment for Capparis scortechinii var. ruthiae

XML Treatment for Capparis trinervia var. chungiana

## Supplementary materials

Supplementary material 1 Figure S1–S8 Data type map, plate and botanical drawing Explanation note Distribution and description of *Cappariskenaboiensis*, Capparisscortechiniivar.ruthiae, *Capparistrinervia var. chungiana* https://binary.pensoft.net/file/642016This dataset is made available under the Open Database License (http://opendatacommons.org/licenses/odbl/1.0/). The Open Database License (ODbL) is a license agreement intended to allow users to freely share, modify, and use this Dataset while maintaining this same freedom for others, provided that the original source and author(s) are credited.Avelinah Julius
