# Supplementary figures and images for: ﻿Capparis (Capparaceae) in Peninsular Malaysia, including a new species and two new varieties
Source: PhytoKeys. 2022 Feb 4;189:99–127. doi: 10.3897/phytokeys.189.49367 (PMC8837500; doi:10.3897/phytokeys.189.49367)

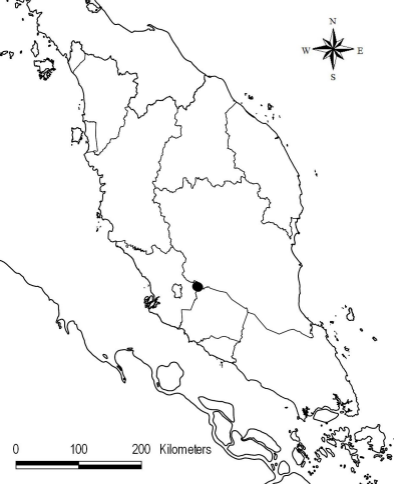

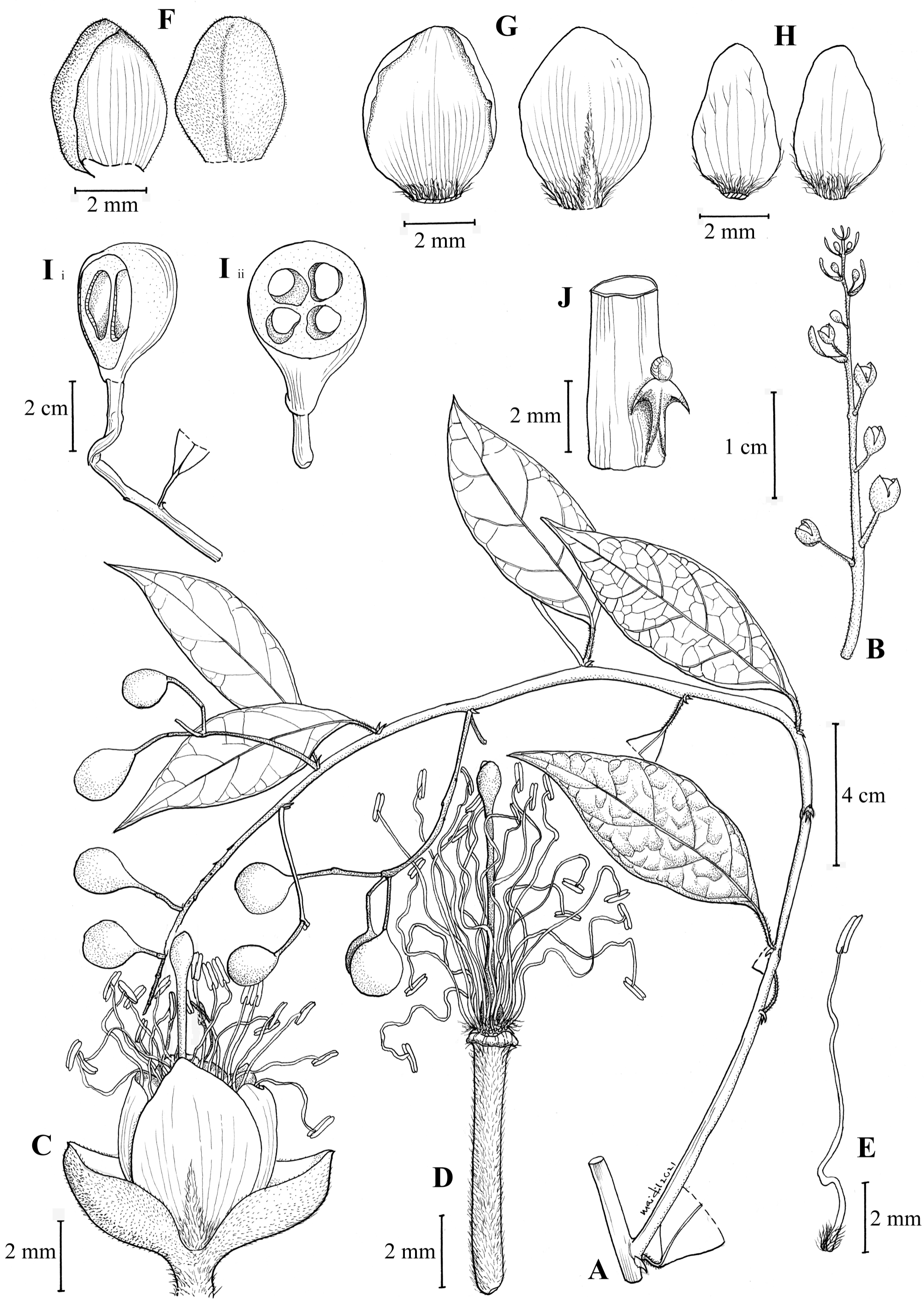

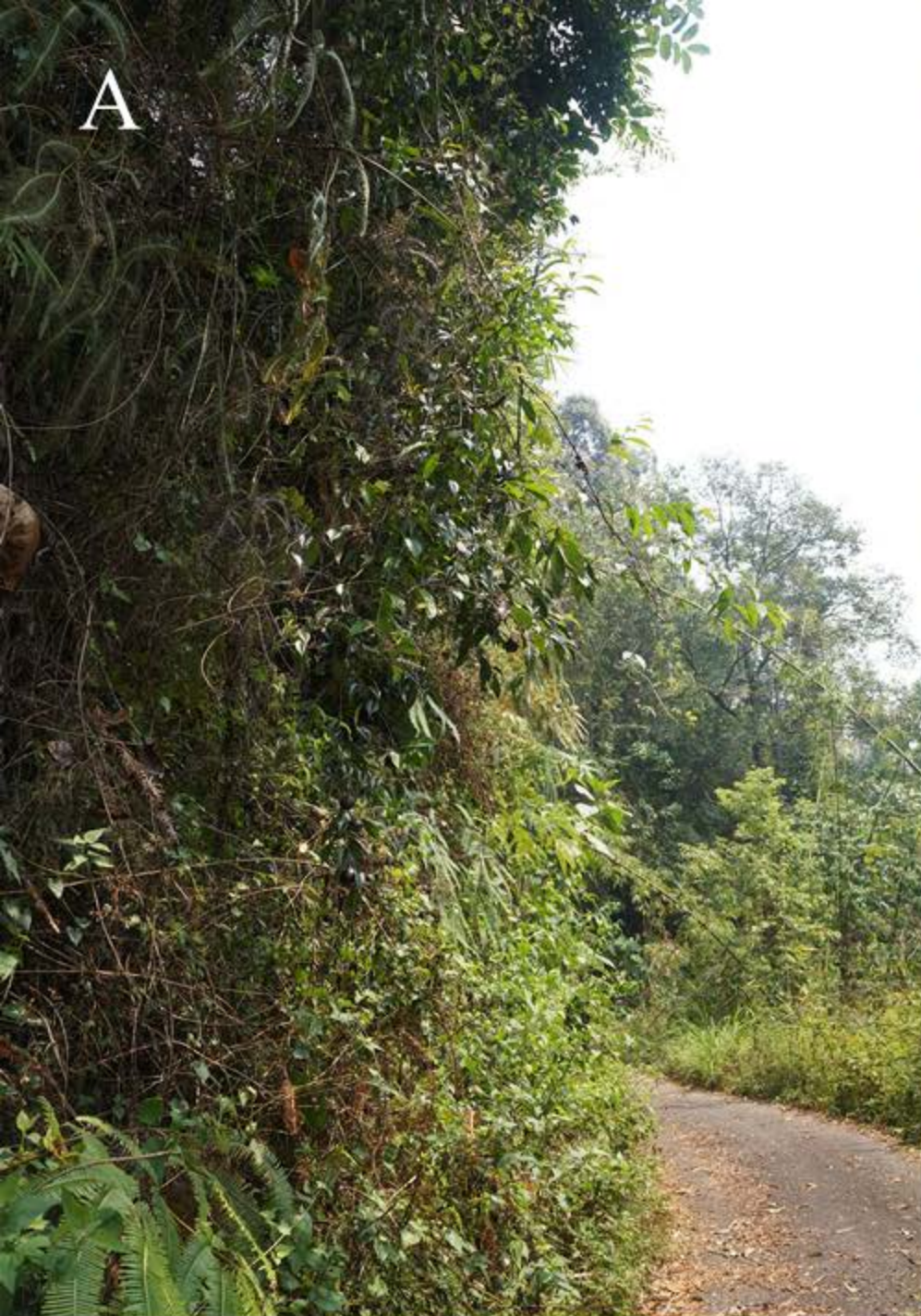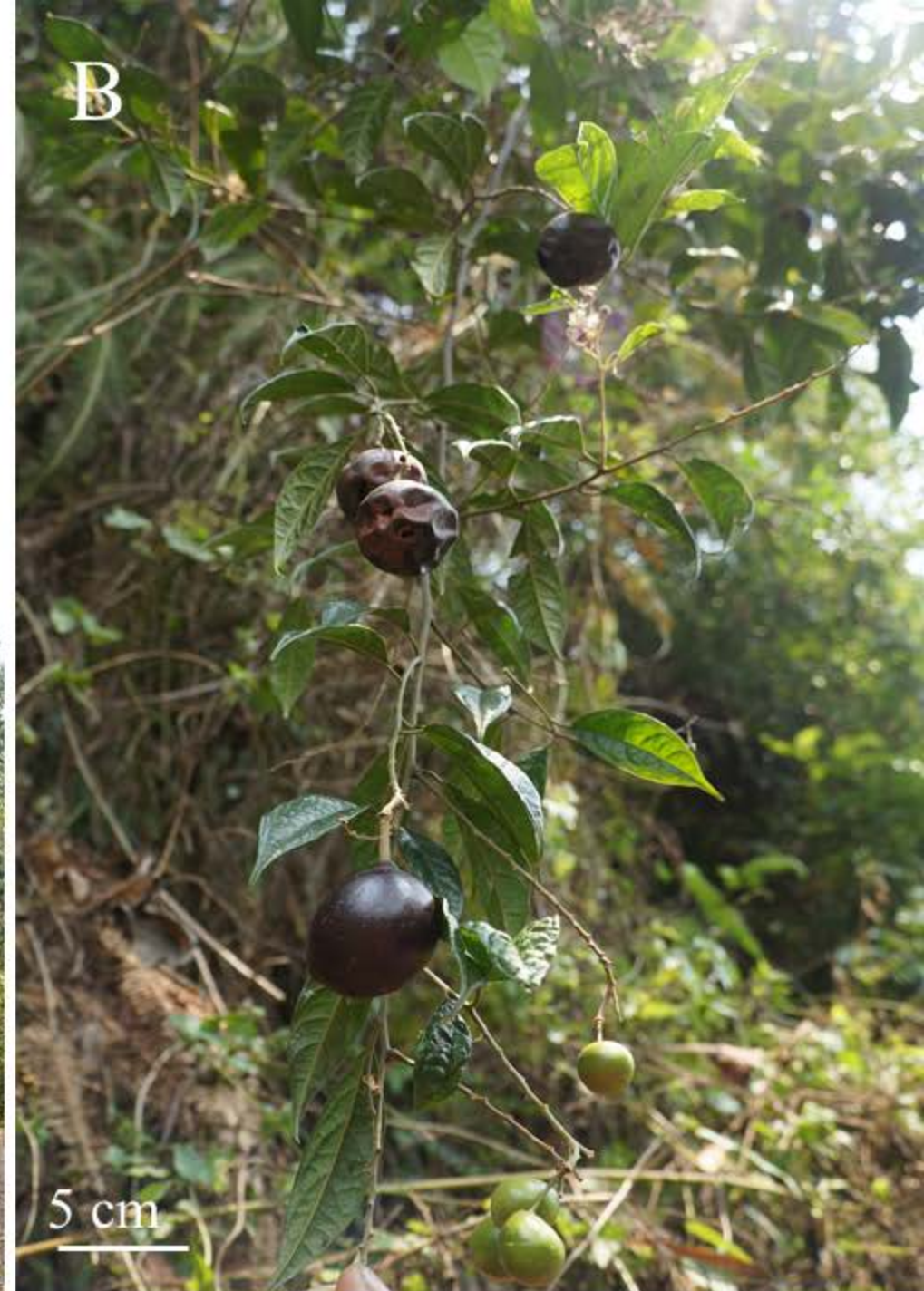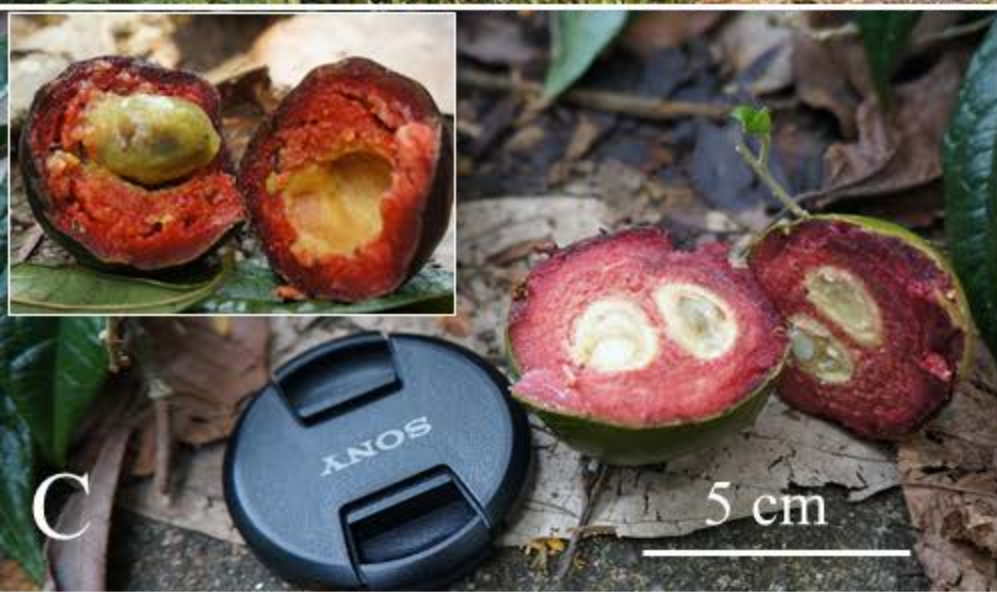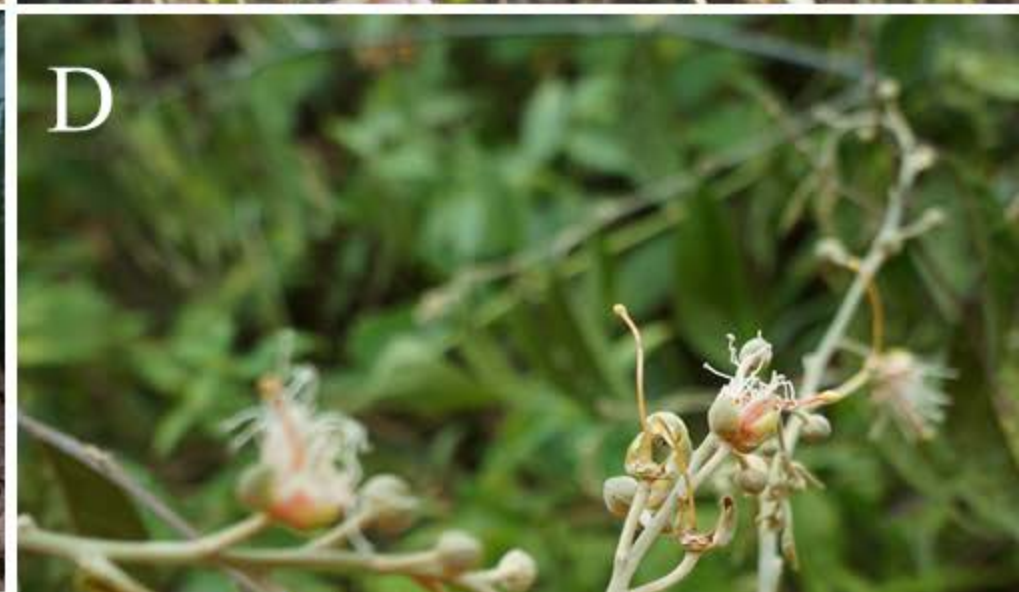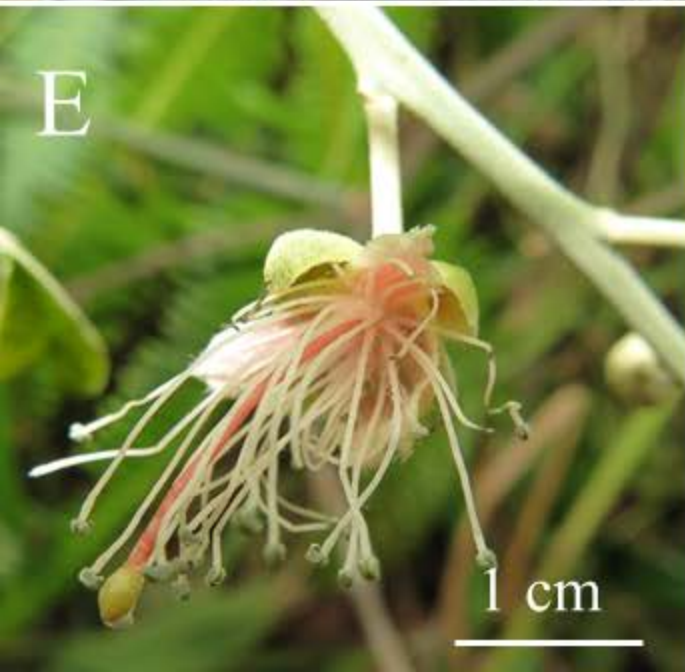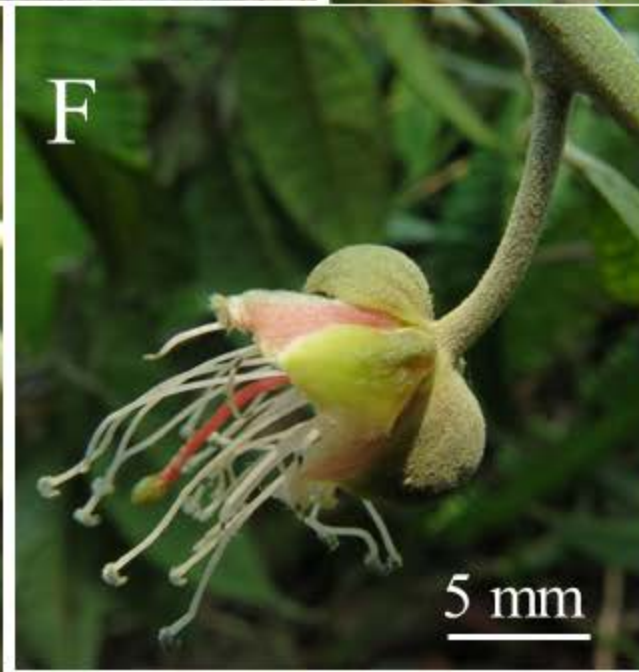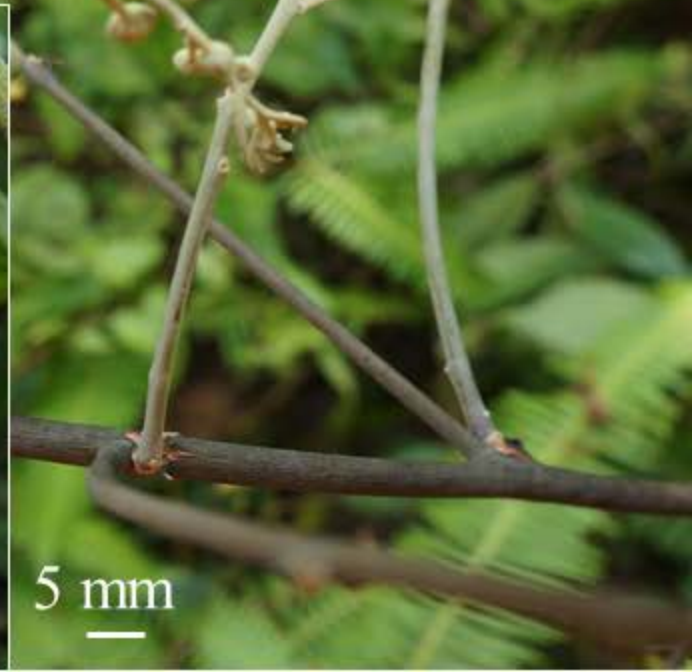

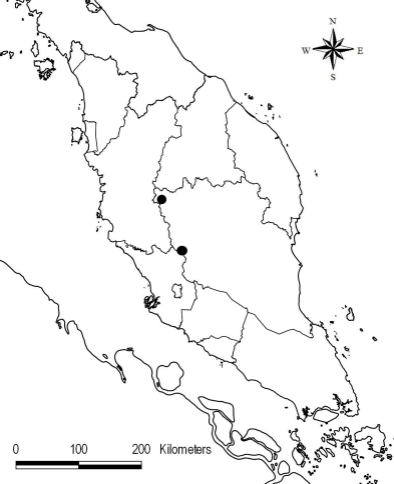

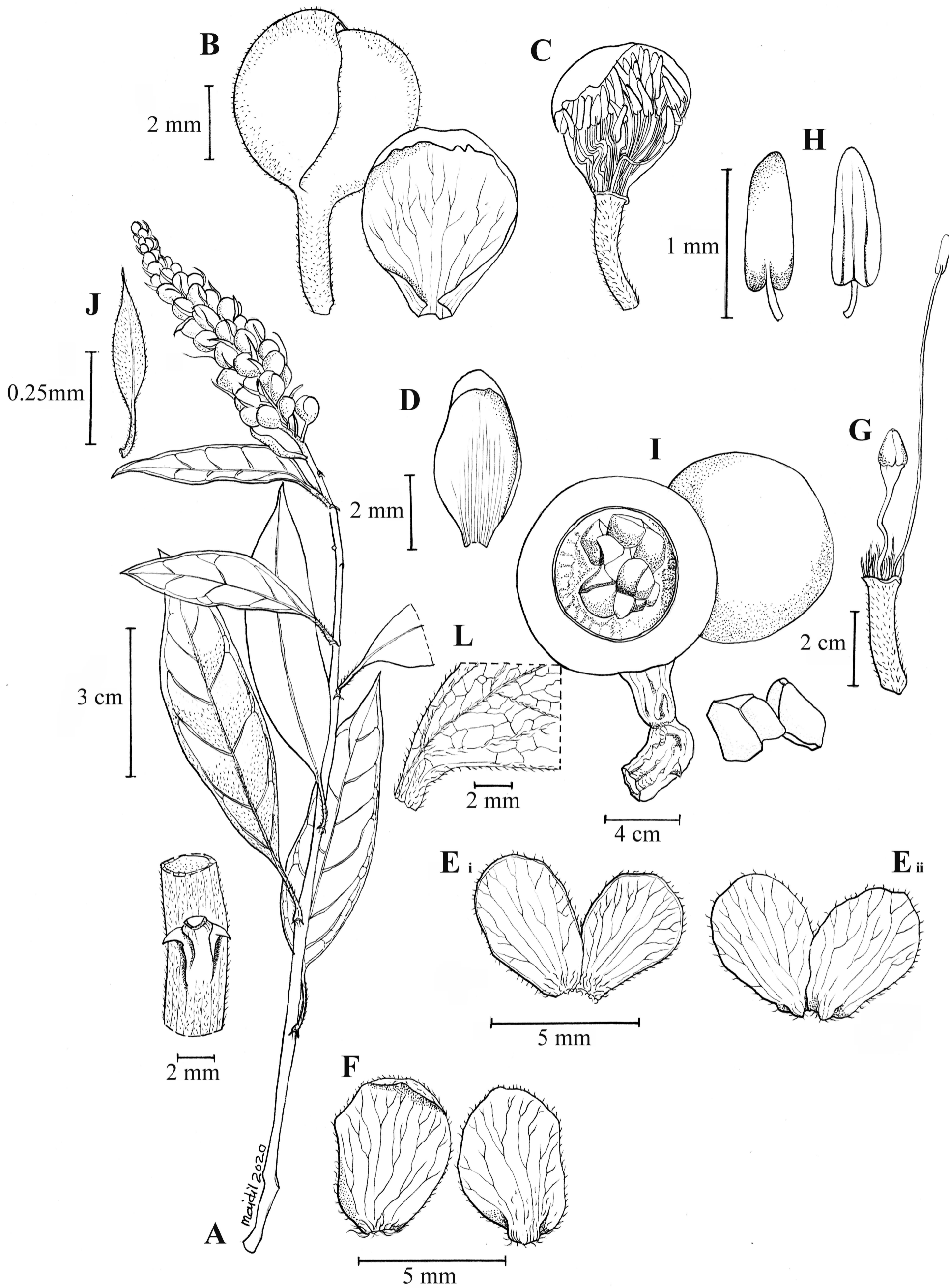

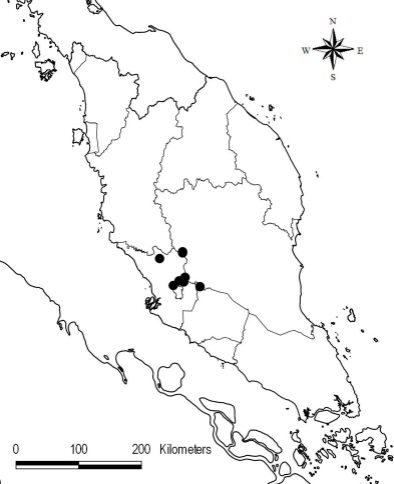

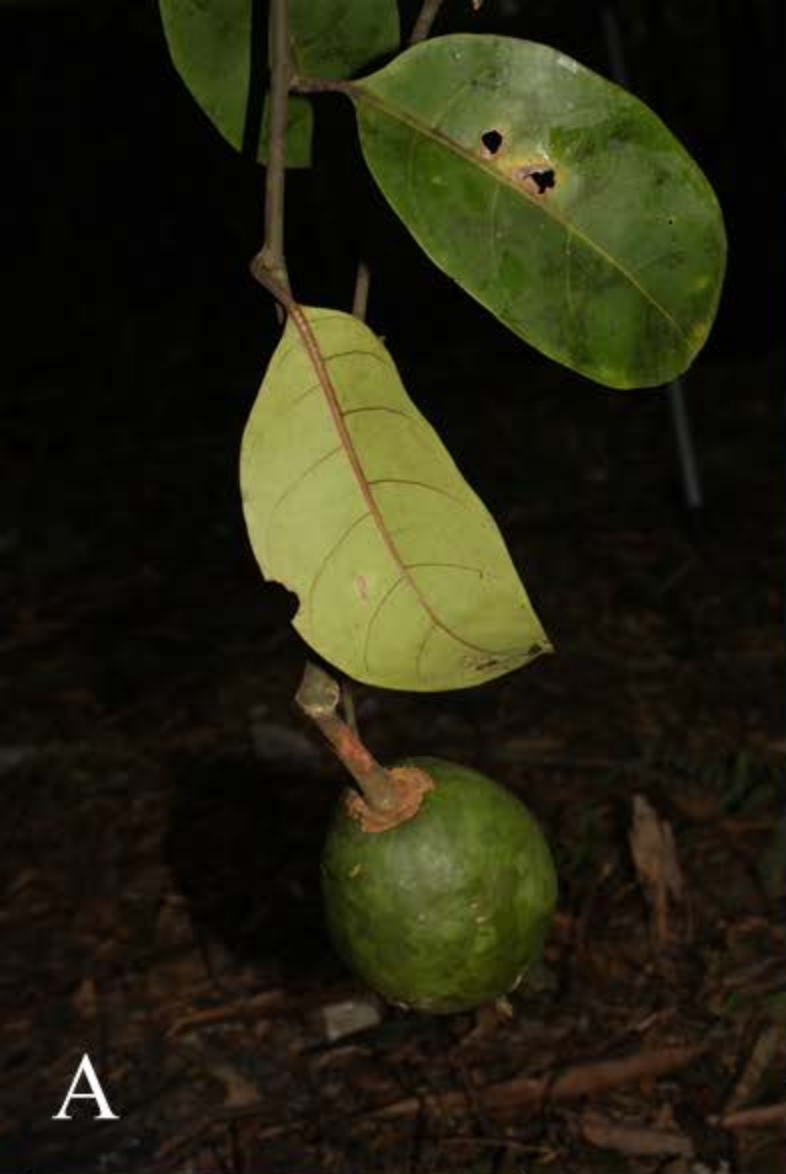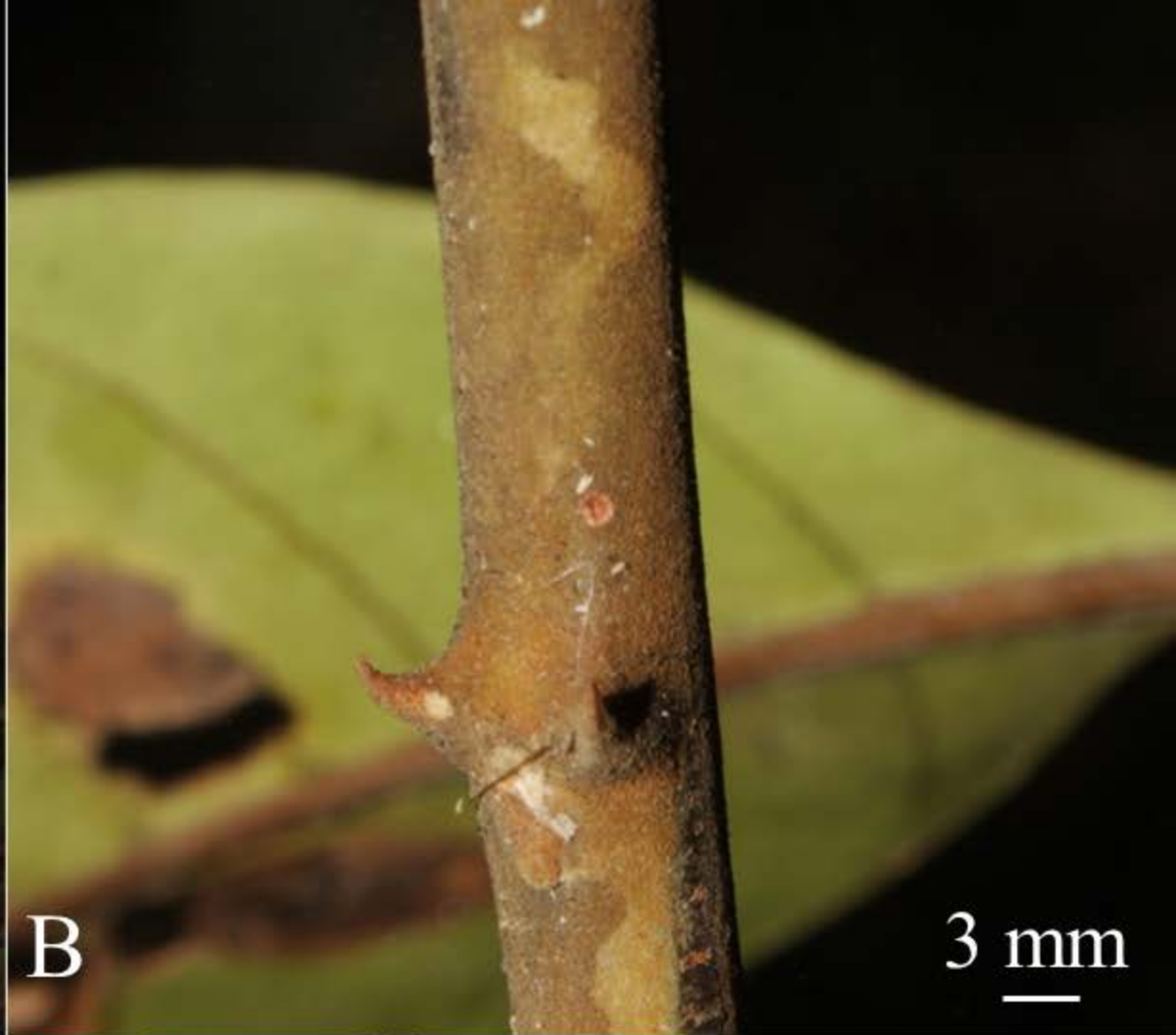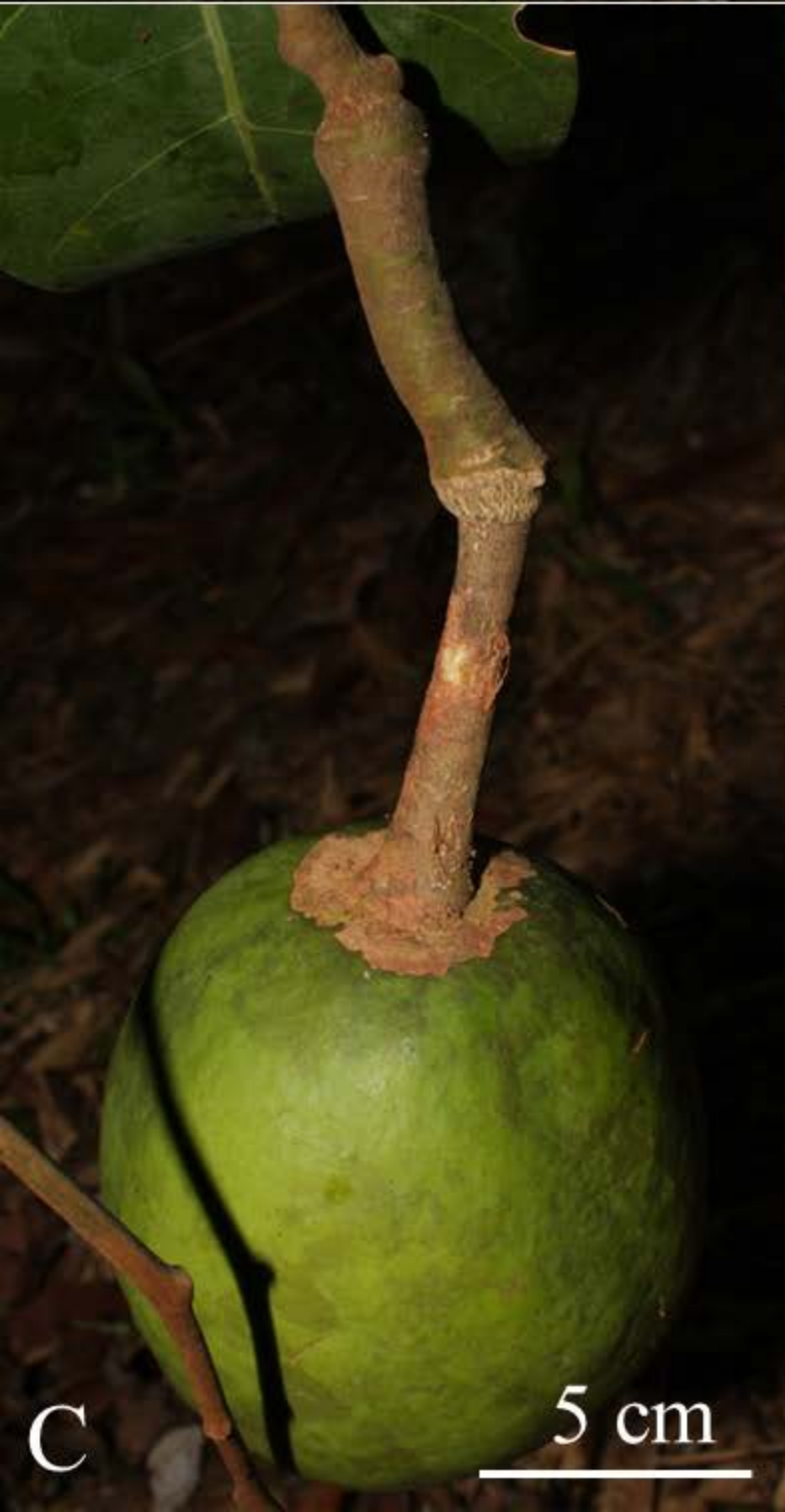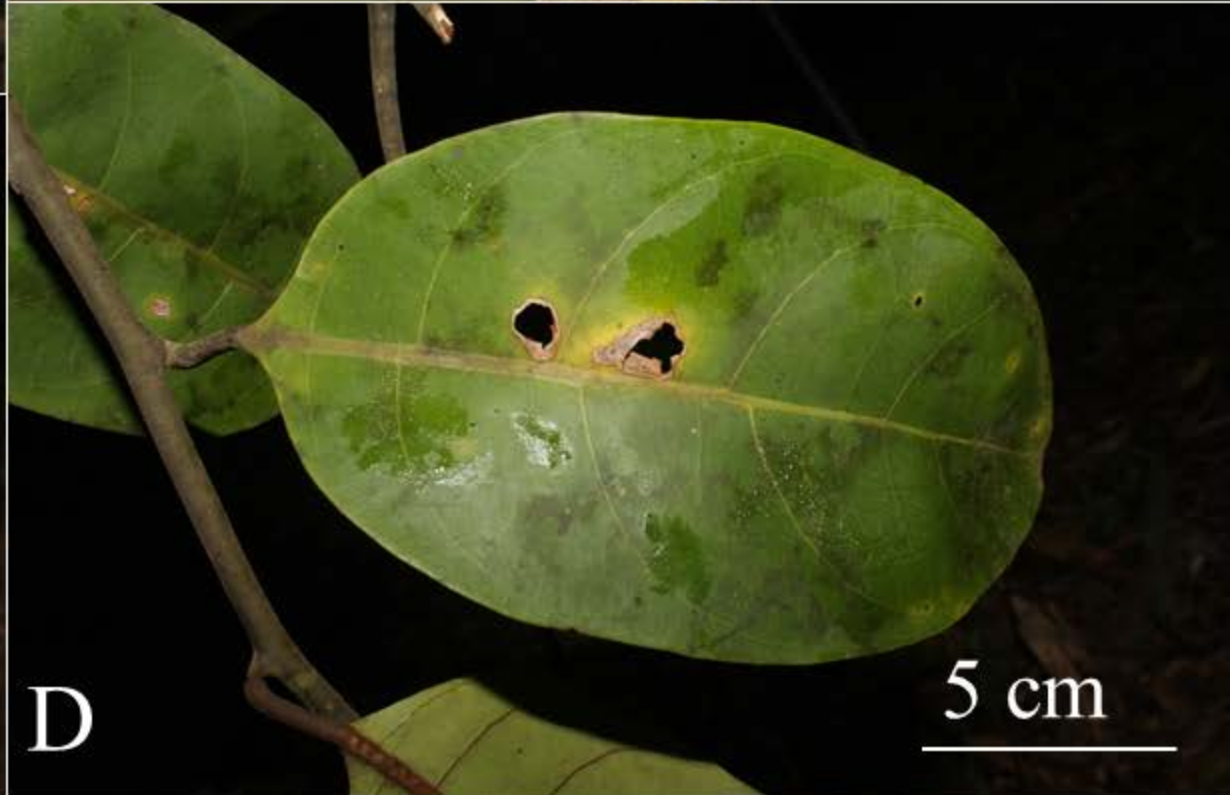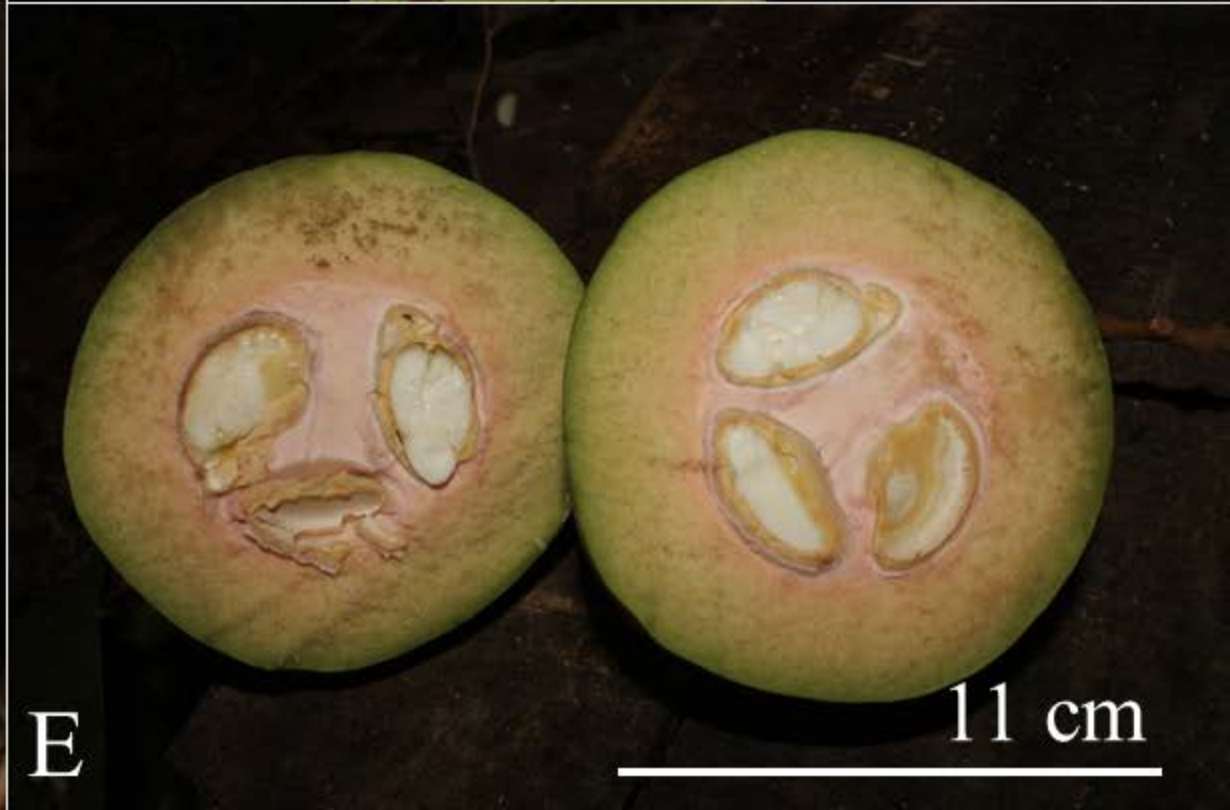

Supplement: Supplementary material 1 — Figure S1–S8 [file phytokeys-189-099-s001.pdf]
